# Multifaceted role of *TRIM28* in health and disease

**DOI:** 10.1002/mco2.790

**Published:** 2024-11-11

**Authors:** Mazaher Maghsoudloo, Khatere Mokhtari, Behdokht Jamali, Amir Gholamzad, Maliheh Entezari, Mehrdad Hashemi, Junjiang Fu

**Affiliations:** ^1^ Key Laboratory of Epigenetics and Oncology the Research Center for Preclinical Medicine Southwest Medical University Luzhou Sichuan China; ^2^ Department of Cellular and Molecular Biology and Microbiology Faculty of Biological Science and Technology University of Isfahan Isfahan Iran; ^3^ Department of Microbiology and Genetic Kherad Institute of Higher Education Busheher Iran; ^4^ Farhikhtegan Medical Convergence Sciences Research Center Farhikhtegan Hospital Tehran Medical Sciences Islamic Azad University Tehran Iran; ^5^ Department of Genetics Faculty of Advanced Science and Technology Tehran Medical Sciences Islamic Azad University Tehran Iran

**Keywords:** cancer pathogenesis, cancer therapy, DNA damage response, transcriptional regulation, *TRIM* family, *TRIM28*

## Abstract

The *TRIM* (tripartite motif) family, with *TRIM28* as a key member, plays a vital role in regulating health and disease. *TRIM28* contains various functional domains essential for transcriptional regulation, primarily through its interaction with *KRAB‐ZNF* proteins, which influence chromatin remodeling and gene expression. Despite extensive research, the precise mechanisms by which *TRIM28* impacts health and disease remain elusive. This review delves into *TRIM28*’s multifaceted roles in maintaining health, contributing to a variety of diseases, and influencing cancer progression. In cancers, *TRIM28* exhibits a dual nature, functioning as both a tumor promoter and suppressor depending on the cellular context and cancer type. The review also explores its critical involvement in processes such as DNA repair, cell cycle regulation, epithelial‐to‐mesenchymal transition, and the maintenance of stem cell properties. By uncovering *TRIM28*’s complex roles across different cancers and other diseases, this review underscores its potential as a therapeutic target. The significance of *TRIM28* as a versatile regulator opens the door to innovative therapeutic strategies, particularly in cancer treatment and the management of other diseases. Ongoing research into *TRIM28* may yield key insights into disease progression and novel treatment options.

## Graphical abstract

The role of TRIM28 in health and disease. This review demonstrated the role of TRIM28 in health and diseases specially cancers. It explored the vital role of *TRIM28* in epigenetics, immune processes, and cancer therapy. This figure was created using BioRender and Microsoft Publisher 2016. HBV, hepatitis B virus; HCV, hepatitis C virus.

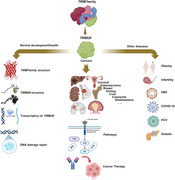

## 1 INTRODUCTION

The proteins encoded by the tripartite motif (*TRIM*) gene family are diverse and involved in a range of biological processes. These proteins comprises a coiled‐coil region, one or two B‐box motifs, and an extremely intriguing new gene (RING) domain—collectively called the RING finger, B‐box, and coiled‐coil (RBCC) motif 1—is present in these proteins.^1^

The *TRIM* superfamily of proteins is distinguished by the remarkably conserved sequence of domains within the RBCC motif. This conservation across different species highlights the RBCC motif as the key defining feature of the superfamily. Even in *TRIM* family members that lack one of the RBCC domains, the remaining domains maintain their order and spacing, further emphasizing the motif's critical role.^2^ Due to the pivotal role of the *TRIM* family in posttranslational protein modification, cellular signaling pathways, and gene regulation, it is unsurprising that different members of the *TRIM* family exhibit a range of effects on cell behavior. These impacts encompass the control of cell multiplication, movement, and infiltration.^3^, ^4^

As noted, the *TRIM* family is extensive, with each member contributing uniquely to both disease and health. Mutations in specific *TRIM* genes, such as *MID1*, *TRIM32*, and *TRIM37*, are linked to hereditary genetic disorders, including X‐linked Opitz G/BBB syndrome, limb‐girdle muscular dystrophy type 2H, and Mulibrey nanism, respectively. Many TRIM proteins respond to interferon (*IFN*), with some serving as downstream effectors in innate immune responses to retroviruses and other viral infections. For example, TRIM proteins exhibiting anti‐HIV activity can disrupt various stages of the virus's life cycle, including preintegration (e.g., *TRIM5*), transcription (e.g., *TRIM22*, *TRIM32*), and assembly (e.g., *TRIM22*, *TRIM15*). Additionally, other *TRIM* genes—such as *TRIM21*, *TRIM25*, *TRIM27*, *TRIM30*, and *TRIM32*—function downstream of *IFN* and pathogen‐recognition receptors, playing a role in modulating innate immune responses to bacterial and viral infections through the activation of *IRF3*, *IRF7*, and *NF‐κB*. Furthermore, *TRIM21* and *TRIM68* are significant targets of autoantibodies in individuals with autoimmune diseases, including systemic lupus erythematosus and Sjögren's syndrome.^5^

We focused our investigation on *TRIM28* due to its significant involvement in various diseases. Addressing this issue is crucial because cancer is one of the main causes of death. In this paper, we not only conducted a bioinformatics analysis and examined the relationship between *TRIM*28 and cellular processes in health and disease, particularly in cancer, but also provided a valuable update on this subject.

## 2 NORMAL DEVELOPMENT/HEALTH OF *TRIM28*

*TRIM28* is an important protein for normal development and health. It is essential in embryonic development, cell differentiation, genomic stability, and the maintenance of adult tissue homeostasis.^6^ *TRIM28* regulates genes associated with pluripotency, ensuring that embryonic stem cells (ESCs) maintain their pluripotent state until differentiation is properly signaled. It also maintains epigenetic marks, such as DNA methylation, which are essential for stable gene silencing during differentiation. Additionally, *TRIM28* contributes to genomic stability by silencing transposable elements (TEs) and repairing double‐strand breaks. Moreover, it is extremely important in brain development, notably in the regulation of neural progenitor cells. *TRIM28* also regulates immune system genes and maintains cellular homeostasis in adult tissues by controlling gene expression and protecting against genomic instability. Furthermore, it plays a role in metabolic processes, such as adipogenesis, which controls the balance between energy storage and expenditure.^7^

### 2.1 The structure and function of *TRIM* family

A RING finger domain, one or more B‐box domains, and a coiled‐coil region are the three primary motifs that make up the *TRIM* family. These motifs, typically located near the protein's N‐terminal, form the tripartite motif that gives the family its name. The RING finger domain, a type of zinc finger motif, binds zinc ions through conserved histidine and cysteine residues. This domain is crucial for the E3 ubiquitin ligase activity of many TRIM proteins, enabling them to transfer ubiquitin molecules to substrate proteins, marking them for degradation by the proteasome. Zinc‐binding motifs are also known as B‐box domains, and TRIM proteins typically contain one or two of these domains (B‐box 1 and B‐box 2). They play roles in protein–protein interactions (PPIs) and stabilization of the TRIM protein structure. The formation of higher‐order oligomers or homo‐ or hetero‐dimers is facilitated by the coiled‐coil region. Since dimerization or oligomerization frequently influences the biological activity of TRIM proteins, this region is essential to their function. TRIM proteins’ C‐terminal region exhibits greater variability, containing different domains depending on the specific protein. These domains include PRY/SPRY, COS, FIL, MATH, and B30.2, which are involved in PPIs, fibronectin type III‐like functions, associations with intermediate filaments, PPIs, and recognition of specific protein substrates, respectively. Furthermore, this protein family plays a role in a number of biological processes, such as cancer, immunological response, cell division and proliferation, ubiquitination, and protein degradation.^8^, ^9^

### 2.2 The structure and function of *TRIM28*

*TRIM28*, along with three other TRIM proteins—TRIM24, TRIM33, and TRIM66—constitutes the *TIF1* family.^10^, ^11^ The domains located in the N‐terminus of *TRIM28* are known as the RBCC domain, also referred to as the *TRIM* domain.^9^ The RING finger domain is distinguished by its cysteine‐rich sequence, being essential to its interaction with the Kruppel‐associated box (KRAB) domain, which is present in many KRAB‐zinc finger (KRAB‐ZFP).^12^, ^13^

The core segment of the TRIM*28* protein harbors the PxVxL pentapeptide domain, facilitating its binding with *HP1*.^14^ Furthermore, akin to other TIF1 proteins, *TRIM28* also possesses the characteristic TSS at its central region, comprising a 25‐amino acid motif abundant in tryptophan and phenylalanine residues. At the carboxyl (C) terminus of *TRIM28*, the plant homeodomain (*PHD*) finger and bromodomain reside, serving to recruit components of NuRD, including the histone deacetylase complex and the *H3K9*‐specific methyltransferase *SETDB1*, thereby promoting chromatin condensation. Both the *PHD* finger and the RING domain, located here, exhibit a structure rich in cysteine and histidine residues, featuring a consensus Cys4–His–Cys3 motif spanning 50–80 residues. The collective presence of the *PHD*, bromodomain, and PxVxL domain is proposed to augment the formation of condensed heterochromatin, marked by increased affinity for *HP1* binding.^14^ *TRIM28* is subject to regulation at multiple levels, encompassing gene transcription, posttranscriptional translation, and posttranslational modifications. For instance, *WDR4* is capable of inducing the transcription of the *TRIM28* gene.^15^ *ZBRK1/ZNF350* inhibits *TRIM28* transcription by repressing the activity of its promoter.^16^ *TRIM28*’s suppressive function involves recruiting *SETDB1* and the *NuRD* complex, as well as interacting with the HP1 protein. Notably, *TRIM28* also acts as an intramolecular E3 *SUMO* ligase.^17^ *TRIM28* possesses ubiquitin E3 ligase activity, facilitating substrate degradation, and enhances the SUMOylation of substrates. SUMOylation is another key post‐translational modification that involves the addition of a small ubiquitin‐like modifier.^18^ Like ubiquitination, *SUMOylation* involves a cascade of enzymatic steps, including E1 activation, E2 conjugation, and E3 ligation, to attach *SUMO* molecules to target substrates.^18^ Although SUMO E3 ligases are not essential for the *SUMOylation* process, their dysregulation can significantly impact substrate *SUMOylation*, contributing to both normal and cancer development.^19^ For normal development, proper SUMOylation is essential. In order to control gene expression, signaling pathways, and developmental processes, SUMO E3 ligases are essential. Because improper SUMOylation can interfere with cellular differentiation, proliferation, and tissue formation, dysregulation of SUMOylation resulting from aberrant E3 ligase function can cause developmental defects.^20^ The transcriptional repression activity of *TRIM28* is dependent on the SUMOylation of three specific lysine residues (K554, K779, and K804). Importantly, within the *TRIM28* molecule, the PHD and bromodomain regions act as SUMO E3 ligases, with the PHD domain binding to UBC9 (the SUMO E2 enzyme) and working together to enhance the *SUMOylation* of *TRIM28*.^21^

### 2.3 *TRIM28* and transcriptional coregulation

Being a transcriptional corepressor, the TRIM28 protein is essential for *KRAB‐ZNF* proteins to perform their repressive function.^22^ In brief, *KRAB*‐*ZNF* proteins, anchored by their zinc finger domains to specific DNA recognition motifs, recruit the TRIM*28* protein. *TRIM28* then serves as a scaffold for various heterochromatin‐inducing factors.^23^ Subsequently, the PHD‐mediated SUMOylation of the bromodomain occurs, which then assists in recruiting *SETDB1* and *NuRD* complex proteins. This sequential process ultimately leads to histone deacetylation.^21^ Furthermore, the HP1 protein interacts with *TRIM28* at the PxVxL motif and associates with the H3K9me3 mark, thereby enhancing the stability of the *TRIM28*‐containing complex that is bound to *KRAB*‐*ZNF*.^24^ Changes in chromatin organization, prompted by *TRIM28* recruitment to particular genomic loci through synthetic constructs, result in the suppression of transcription from RNA polymerase I, II, and III promoters.^25^ *TRIM28* plays a role in stabilizing the pausing of RNA polymerase II near the transcriptional start site in various inactive genes.^26^ The modulation of Pol II pausing is contingent upon the phosphorylation status of *TRIM28*. When *TRIM28* is phosphorylated at *Ser824*, it facilitates the release of Pol II from pausing, leading to the rapid transcription of target genes. Furthermore, studies have shown that *TRIM28* regulates the transcription of a subset of lncRNAs in mammalian cells through a similar mechanism involving Pol II pausing at their transcriptional start sites.^26^ Given the abundance of noncoding RNAs encoded by mammalian genomes, which surpass mRNA genes by a significant margin, it is reasonable to speculate on the extensive role of *TRIM28* in the regulation of genome‐wide transcription.^27^ Figure S1 represents the PPI between *TRIM28* and other proteins. The strong interaction between *TRIM28* and the *ZNF* and *CBX* families is seen in Figure S1. The data indicate a robust association between members of the *ZNF* family and *TRIM28*, an essential transcriptional regulator implicated in DNA binding and regulation of gene expression. This interaction is particularly relevant in the context of both normal development and disease, particularly in cancer since dysregulation of *ZNF* proteins has been related to carcinogenesis through their effect on tumor suppressor genes and oncogenes. *TRIM28* is essential for the creation and preservation of epigenetic marks when it forms a complex with ZNF proteins. It plays a role in the processes that preserve genomic stability by forming heterochromatin and silencing TEs. Normal cellular differentiation and the expression of genes specific to particular tissues during development depend on this epigenetic regulation. Diseases and disorders related to development can result from disruptions in these processes.^7^

Significant ties to the Chromobox (*CBX*) family, which are crucial components of the polycomb repressive complexes that maintain the repression of gene transcription, are also highlighted in the image. Because they maintain tumor suppressor genes in their repressed form, members of the *CBX* family are essential to the initiation and progression of cancer. These interactions demonstrate how essential *TRIM28* is for regulating a network of protein partners that improve gene silencing and chromatin remodeling. This draws attention to the role of the protein in biological processes like differentiation and development, as well as any possible links to cancer. *TRIM28* may affect the epigenetic landscape of cancer cells by controlling the relationships between various protein families, so fostering an environment that is favorable to malignancy and resistance to treatment.

### 2.4 *TRIM28* and DNA damage repair

One of the many cytotoxic DNA lesions that can trigger the cellular response to DNA damage is double‐strand breaks. Once DNA damage is detected, *TRIM28* aids in chromatin remodeling near the break location. This is accomplished by *TRIM28* through the use of histone‐modifying enzymes, including HDACs.^28^ Additionally, the activation of ataxia telangiectasia mutated (ATM) kinase, belonging to PIKK family, is essential for addressing this form of damage.^29^ The *ATM* kinase phosphorylates the TRIM*28* protein at *Ser824* located within the C‐terminus and at *Ser473* in close proximity to the *HP1* binding domain.^30^, ^31^ The process of *TRIM28* phosphorylation marks one of the initial stages in the cellular response to DNA damage.^31^ Elimination of the *Ser824* phosphorylation site on *TRIM28* results in the inability to induce chromatin decondensation in response to double‐strand breaks within DNA. Additionally, it increases the cells’ susceptibility to agents that induce double‐strand breaks.^30^ Reducing *TRIM28* levels or introducing a constitutive *Ser824* phosphorylation mutation (*TRIM28*–*S824D* mutant) results in persistent chromatin relaxation. These results underscore the importance of chromatin relaxation as a core mechanism in the DNA damage response and highlight its key mediators.^30^ As evidenced in melanoma cancer cells, the *MAGE*‐*C2* protein has the capacity to trigger *ATM*‐dependent phosphorylation of *TRIM28* at *Ser824*, thereby favoring DNA damage repair mechanisms over apoptosis.^32^ *MAGEC2* enhances the coprecipitation of *TRIM28* with *ATM*, which is essential for the increased phosphorylation of *TRIM28*. This heightened phosphorylation level, in turn, enhances DNA damage repair, thereby promoting tumor progression.^32^ *TRIM28* operates in inhibiting E2F transcription factor 1 (*E2F1*) and may act as a partial backup mechanism to impede *E2F1*‐induced apoptosis in cancer cells.^33^

*TRIM28* has been linked to the regulation of genes involved in neuronal development and function. Accumulation of DNA damage in neurons, a hallmark of neurodegenerative diseases such as Huntington's and Alzheimer's, can be facilitated by abnormalities in *TRIM28* activity.^34^ *TRIM28* interacts with RE‐1‐silencing transcription factor and huntingtin protein mutated, resulting in changes in transcriptional repression and chromatin remodeling, which could be an effective factor in increasing DNA damage and neurodegeneration.^35^

## 3 THE ROLE OF *TRIM28* IN DISEASES

Although the significance of epigenetics was initially recognized for its role in tissue development, growing evidence now indicates that it is equally crucial in the onset and progression of numerous common diseases. Numerous novel risk factors have been discovered through population‐based epigenetic epidemiological studies examining the impact of epigenetic changes on common diseases; however, this relatively nascent field continues to confront several distinct challenges. Among these, *TRIM28* has been identified as a pivotal gene due to its regulatory influence on epigenetic processes. Variations in the expression or function of *TRIM28* can aid in the development of a wide variety of illnesses, underscoring its vital importance in both health and disease contexts.

### 3.1 The *TRIM* family in cancer pathogenesis

**FIGURE 1:**
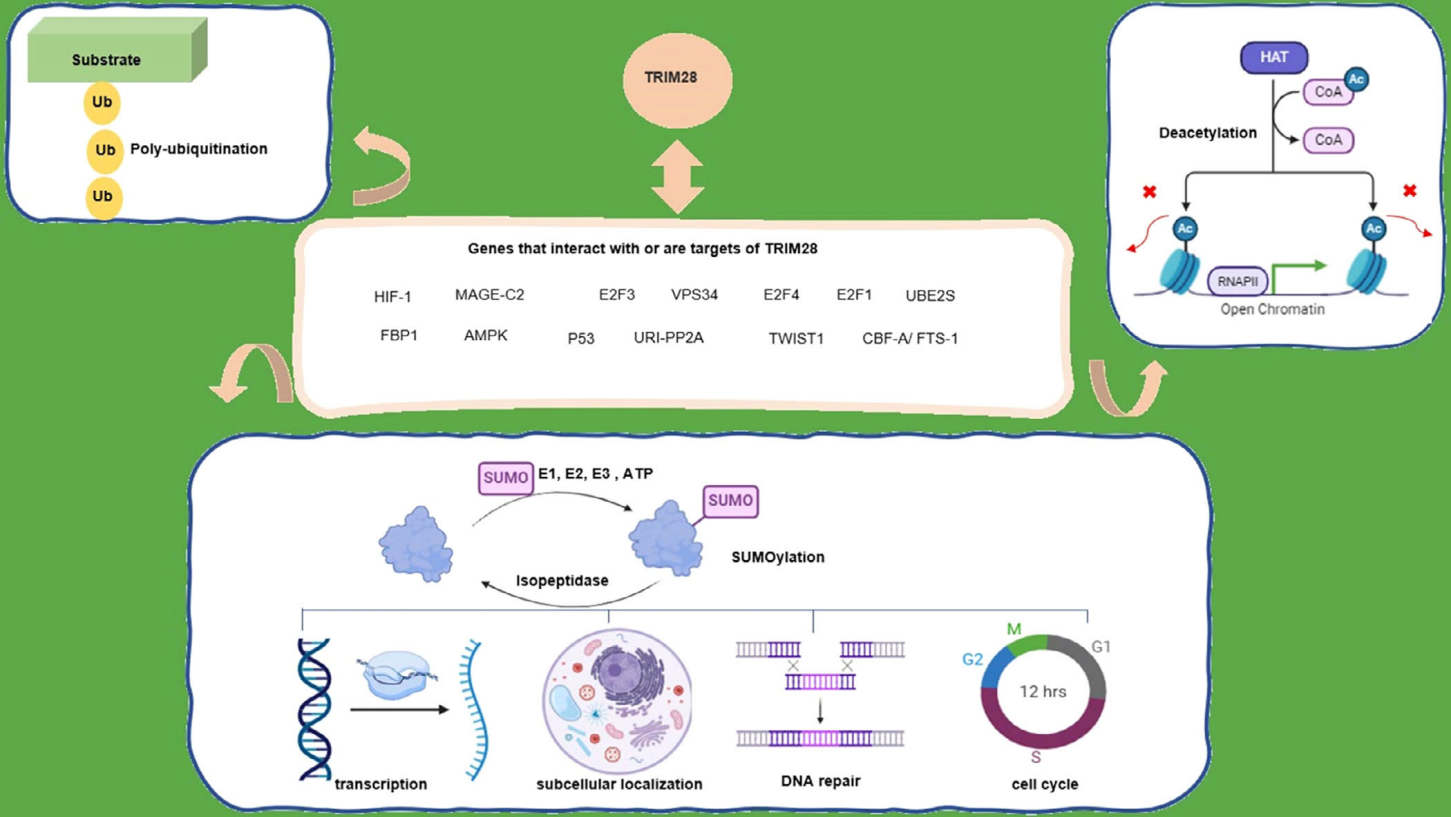
Genes that interact with or are targets of *TRIM28*. This figure represents the interaction between *TRIM28* and target genes, along with their effects, functions, and modifications, including polyubiquitination, deacetylation, and SUMOylation. This figure was created using BioRender and Microsoft Publisher 2016.

Numerous genes within the *TRIM* family exhibit notable modifications across different cancer types.^3^, ^4^ TRIM family proteins associated with some various cancers are reported in Table 1. In specific cases, the expression levels of *TRIM*s can function as biomarkers and prognostic indicators for cancer.^36^ *KRAB*‐associated protein 1 (a large multidomain protein (110 kDa), *KAP1*, also referred to as *TRIM28* and *TIF1b*), initially garnered significant interest in 1996.^37^ Subsequently, extensive research has been conducted on the involvement of *TRIM28* in various facets of cellular biology, resulting in the elucidation of the intricate characteristics of the TRIM*28* protein. Notably, one of the primary pathways in which this pivotal gene exerts its influence is cancer. Table 2 shows a list of proteins that interact with or target by *TRIM28* in cancers (Figure 1).

**TABLE 1:** *TRIM* family proteins associated with various cancers.

TRIM family	AML	HCC	CRC	Squamous carcinoma	Lung carcinoma	Glioblastoma	Pancreatic carcinoma	Ovarian cancer	Breast carcinoma	NPC	Kidney cancer	Melanoma	Gastric carcinoma	HEC	Testicular cancer	Thyroid cancer	Cervical cancer	Prostate cancer	HNSCC	Osteosarcoma	References
*TRIM1*			^*^						^*^		^*^										^38^, ^39^, ^40^
*TRIM2*		^*^	^*^	^*^	^*^		^*^	^*^	^*^		^*^	^*^					^*^		^*^	^*^	^38^, ^41^, ^42^, ^43^, ^44^
*TRIM3*		^*^	^*^		^*^	^*^		^*^	^*^				^*^	^*^		^*^	^*^				^9^, ^39^, ^41^, ^45^, ^46^, ^47^, ^48^, ^49^
*TRIM4*		^*^							^*^											^*^	^50^, ^51^, ^52^
*TRIM5*		^*^																	^*^		^43^, ^53^
*TRIM6*		^*^	^*^		^*^				^*^												^38^, ^54^, ^55^, ^56^
*TRIM7*		^*^			^*^	^*^					^*^		^*^						^*^	^*^	^9^, ^43^, ^57^, ^58^, ^59^, ^60^, ^61^
*TRIM8*			^*^	^*^	^*^	^*^		^*^	^*^		^*^			^*^			^*^			^*^	^39^, ^41^, ^62^, ^63^, ^64^, ^65^
*TRIM9*			^*^		^*^				^*^					^*^							^39^, ^66^, ^67^
*TRIM10*	^*^	^*^																		^*^	^9^, ^68^
*TRIM11*		^*^		^*^	^*^	^*^		^*^	^*^	^*^			^*^	^*^		^*^	^*^	^*^	^*^	^*^	^39^, ^41^, ^43^, ^54^, ^69^, ^70^, ^71^, ^72^, ^73^, ^74^, ^75^, ^76^, ^77^
*TRIM13*	^*^		^*^		^*^				^*^		^*^								^*^		^38^, ^40^, ^41^, ^43^, ^78^
*TRIM14*	^*^	^*^	^*^		^*^				^*^			^*^	^*^	^*^		^*^	^*^		^*^	^*^	^9^, ^39^, ^41^, ^79^, ^80^, ^81^, ^82^, ^83^, ^84^
*TRIM15*			^*^		^*^								^*^	^*^							^39^, ^41^, ^85^, ^86^
*TRIM16*		^*^		^*^	^*^			^*^	^*^		^*^	^*^	^*^					^*^	^*^		^41^, ^54^, ^87^, ^88^
*TRIM17*	^*^								^*^			^*^	^*^							^*^	^89^, ^90^
*TRIM21*		^*^				^*^		^*^	^*^	^*^	^*^		^*^	^*^		^*^	^*^		^*^	^*^	^41^, ^43^, ^53^, ^91^, ^92^, ^93^, ^94^, ^95^, ^96^, ^97^
*TRIM22*	^*^	^*^	^*^		^*^	^*^		^*^	^*^			^*^	^*^						^*^	^*^	^9^, ^39^, ^41^, ^43^, ^79^, ^98^, ^99^, ^100^, ^101^, ^102^, ^103^
*TRIM23*		^*^	^*^										^*^								^9^, ^104^
*TRIM24*	^*^	^*^	^*^		^*^	^*^		^*^	^*^	^*^	^*^		^*^	^*^			^*^	^*^	^*^		^9^, ^10^, ^39^, ^41^, ^54^, ^105^, ^106^, ^107^, ^108^
*TRIM25*	^*^	^*^	^*^		^*^	^*^		^*^	^*^								^*^	^*^	^*^		^9^, ^41^, ^43^, ^109^, ^110^
*TRIM26*		^*^	^*^			^*^				^*^	^*^					^*^	^*^			^*^	^9^, ^38^, ^40^, ^111^, ^112^, ^113^, ^114^, ^115^
*TRIM27*		^*^	^*^		^*^	^*^		^*^	^*^	^*^	^*^	^*^	^*^	^*^				^*^			^41^, ^116^, ^117^, ^118^
*TRIM28*	^*^	^*^	^*^		^*^	^*^	^*^	^*^	^*^		^*^	^*^	^*^	^*^		^*^	^*^	^*^	^*^	^*^	^10^, ^41^, ^43^, ^54^, ^119^, ^120^, ^121^, ^122^, ^123^
*TRIM29*		^*^	^*^		^*^		^*^	^*^	^*^	^*^		^*^	^*^	^*^	^*^	^*^	^*^	^*^	^*^	^*^	^9^, ^39^, ^41^, ^86^, ^124^
*TRIM31*	^*^	^*^	^*^	^*^	^*^	^*^	^*^	^*^	^*^	^*^	^*^	^*^	^*^		^*^	^*^			^*^		^39^, ^41^, ^54^, ^79^, ^125^, ^126^, ^127^, ^128^
*TRIM32*	^*^	^*^		^*^	^*^				^*^				^*^						^*^		^9^, ^41^, ^53^, ^80^, ^129^, ^130^
*TRIM33*		^*^	^*^		^*^		^*^		^*^		^*^		^*^					^*^	^*^		^9^, ^10^, ^41^, ^131^, ^132^, ^133^
*TRIM35*		^*^	^*^		^*^				^*^												^9^, ^38^, ^134^, ^135^
*TRIM36*					^*^			^*^	^*^				^*^	^*^				^*^			^136^, ^137^, ^138^
*TRIM37*		^*^	^*^		^*^	^*^		^*^	^*^		^*^	^*^	^*^		^*^		^*^			^*^	^41^, ^54^, ^70^, ^139^
*TRIM38*					^*^																^140^
*TRIM39*			^*^					^*^	^*^	^*^											^126^, ^141^, ^142^
*TRIM44*		^*^	^*^		^*^	^*^		^*^	^*^		^*^	^*^	^*^	^*^	^*^	^*^	^*^	^*^	^*^	^*^	^9^, ^40^, ^41^, ^53^, ^143^, ^144^, ^145^, ^146^, ^147^, ^148^, ^149^
*TRIM45*		^*^			^*^	^*^															^54^, ^150^, ^151^
*TRIM46*					^*^				^*^											^*^	^152^, ^153^
*TRIM47*		^*^	^*^		^*^			^*^	^*^		^*^		^*^					^*^			^38^, ^40^, ^41^, ^53^, ^154^, ^155^
*TRIM48*						^*^															^156^
*TRIM50*		^*^						^*^					^*^						^*^		^9^, ^38^, ^41^, ^157^, ^158^
*TRIM52*		^*^			^*^	^*^		^*^													^54^, ^153^, ^159^
*TRIM54*													^*^								^160^
*TRIM55*		^*^	^*^		^*^								^*^						^*^		^9^, ^38^, ^43^, ^161^, ^162^
*TRIM56*	^*^	^*^		^*^	^*^	^*^		^*^	^*^										^*^		^9^, ^43^, ^163^
*TRIM58*	^*^	^*^	^*^		^*^				^*^		^*^		^*^							^*^	^9^, ^41^, ^105^, ^164^, ^165^, ^166^, ^167^
*TRIM59*		^*^	^*^		^*^	^*^		^*^	^*^		^*^	^*^	^*^	^*^			^*^	^*^	^*^	^*^	^41^, ^54^, ^168^, ^169^, ^170^, ^171^, ^172^, ^173^
*TRIM62*	^*^	^*^	^*^		^*^		^*^		^*^								^*^				^9^, ^41^, ^68^, ^174^
*TRIM63*									^*^		^*^					^*^			^*^		^41^, ^43^, ^175^, ^176^
*TRIM65*		^*^			^*^				^*^		^*^		^*^				^*^				^41^, ^177^, ^178^, ^179^, ^180^, ^181^
*TRIM66*		^*^	^*^		^*^	^*^			^*^									^*^		^*^	^10^, ^41^, ^54^, ^169^, ^182^, ^183^
*TRIM67*	^*^				^*^				^*^												^184^
*TRIM68*																		^*^		^*^	^41^, ^185^
*TRIM71*		^*^			^*^			^*^							^*^						^9^, ^186^, ^187^, ^188^
*TRIM72*		^*^							^*^												^53^, ^189^
*TRIM73*			^*^		^*^		^*^		^*^												^39^, ^157^

* indicates *TRIM* family members that have been previously reported in the specific cancer types listed.

*Abbreviations*: AML, acute myelocytic leukemia; CRC, colorectal cancer; HCC, hepatocellular carcinoma; HEC, human esophageal cancer; HNSCC, head and neck squamous cell carcinoma; NPC, nasopharyngeal carcinoma.

**TABLE 2:** The proteins that interact with or target by *TRIM28* in cancer‐related contexts.

		Modification								
Author	Target	Poly‐ubiquitination	Deacetylation	HDAC1‐mediated deacetylation	SUMOylation	None	Effect	Oncogenic	Tumor suppressive	References
*Pineda, Carlos T., et al. 2015*	*AMPK*	^*^					Degradation of *AMPK*	^+^		^190^, ^191^
*Pineda, Carlos T. et al. 2015*										
*Venkov, Christo D., et al. 2007*	*CBF‐A/FTS‐1*					^*^	Upregulation of mesenchymal markers such as *VIM* and *FSP1*	^+^		^192^
*Hu, Chen, et al. 2012*	*E2F1*			^*^			The suppression of *E2F1* activity	^+^		^193^
*Chen, Lu, et al. 2012*	*E2F3, E2F4*			^*^			The deactivation of *E2F3* and *E2F4*		^+^	^194^
*Jin, X., et al. 2017*	*FBP1*	^*^					Degradation of *FBP1*	^+^		^195^
*Okamoto, Koji, et al. 2006*	*P53*	^*^	^*^				Inactivation/degradation of *P53*	^+^		^196^, ^197^
*Wang, Chuangui, et al. 2005*										
*Wei, Chunli, et al. 2016*	*TWIST1*					^*^	The interaction and stabilization of *TWIST1*	^+^		^198^
*Mita, Paolo, et al. 2016*	*URI–PP2A*					^*^	The removal of phosphate groups from *TRIM28*, specifically at the *Ser824* site, leads to the condensation of chromatin		^+^	^199^
*Yang, Yonghua, et al. 2013*	*VPS34 (PI3KC3)*				^*^		Activation of *VPS34*	^+^		^200^
*Zhang, Ren‐Yu, et al. 2021*	*UBE2S*	^*^					Accelerates cell cycle by ubiquitination of *p27*	^+^		^201^
*Yang, Yongkang, et al. 2022*	*HIF‐1*					^*^	Release paused RNA polymerase II	^+^		^202^
*Yao‐Jen Chang, et al. 2023*	*STAT3*	^*^	^*^		^*^		Activation of STAT3 signaling, inflammation and tumorigenesis	^+^		^203^
*Kailang Li, et al. 2024*	*BRCA1*	^*^			^*^		*BRCA1* repression and genomic instability and cancer progression		^+^	^120^

Abbreviations: *AMPK*, AMP‐activated protein kinase; *BRCA1*, breast cancer gene 1; *CBF‐A*, CArG box‐binding factor A; *E2F1*, E2F transcription factor 1; *E2F3*, E2F transcription factor 3; *E2F4*, E2F transcription factor 4; *FBP1*, fructose‐bisphosphatase 1; *FSP1*, fibroblast‐specific protein 1; *FTS‐1*, fibroblast transcription site‐1; *HIF‐1*, hypoxia‐inducible factor 1; *PI3KC3*, phosphatidylinositol 3‐kinase catalytic subunit type 3; *PP2A*, protein phosphatase 2; *Ser824*, serine 824; *STAT3*, signal transducer and activator of transcription 3; *TWIST1*, Twist family bHLH transcription factor 1; *UBE2S*, ubiquitin conjugating enzyme E2 S; *URI*, unconventional prefoldin RPB5 interactor protein; *VIM*, vimentin; *VPS34*, vacuolar protein sorting.

* indicates types of protein modifications that interact with or target by *TRIM28* in various cancers.

^+^ represents types of oncogenic/tumor‐suppressive proteins that interact with or target by *TRIM28* in various cancers.

*TRIM28* not only contributes to the progression of various tumors and worsens their prognosis, as detailed in Table S1, but it also exhibits anticancer properties in certain key cancers, which are presented in Table 3. These contrasting roles highlight the dual nature of *TRIM28*’s involvement in cancer, emphasizing the complexity of its function and the importance of understanding its context‐dependent effects in cancer biology.

**TABLE 3:** Key cancers in which *TRIM28* exhibits antitumor effects

Type of cancer	Function	References
Prostate cancer	*URI* uses *PP2A* phosphatase to control *KAP1* phosphorylation and transcriptional repression.	^199^
Kidney cancer	*TRIM28* facilitates the ubiquitination and degradation of *TFE3*, hence preventing the growth of cells that cause renal cell carcinoma.	^204^
Hepatocellular carcinoma	Hepatocellular carcinoma is prevented from progressing by *HDAC6* through the formation of a transcriptional repression complex with *TRIM28*.	^205^
Colorectal cancer	*TRIM28* interacts with *CARM1* mechanistically, preventing *CARM1* from degrading.	^206^
Lung cancer	*TRIM28* can modulate cell proliferation by mediating interactions between *HDAC1* and *E2F*.	^194^

Abbreviations: *CARM1*; Coactivator‐associated arginine methyl transferase 1, *E2F*; E2 factor, *HDAC1*; Histone deacetylase 1, *HDAC6*; Histone deacetylase 6, *KAP1*; KRAB domain‐associated protein 1, *PP2A*; Protein phosphatase 2A, and *TFE3*; Transcription factor E3.

### 3.2 *TRIM28* and cancer

Currently, the clinical significance of *TRIM28* in numerous diseases remains unclear. Nevertheless, there are documented instances indicating a connection between the level of *TRIM28* expression and cancer. It is worth noting that *TRIM28* is also active during developmental stages, exhibiting notably high expression levels in ESCs as well as various tumor types, a topic that will be explored in greater detail. For instance, elevated expression of the *TRIM28* gene has been linked to metastatic cervical cancer (CC).^207^ Increased expression of the *TRIM28* gene has also been demonstrated in gastric cancer (GC), correlating with unfavorable prognosis ^208^; Overexpression of the *TRIM28* gene has also been identified in the peripheral blood of patients with GC.^207^ The expression level of *TRIM28* was also found to be elevated in ovarian cancer samples, and this elevation was linked to aggressive clinical characteristics.^120^ Moreover, high expression of *TRIM28* served as an independent predictor for ovarian cancer patients.^209^ *TRIM28* is markedly overexpressed in gliomas, and this overexpression is associated with decreased rates of overall and progression‐free survival.^210^ Additionally, *TRIM28* holds promise as a prospective biomarker for predicting tumor classification in glioblastoma.^211^ Moreover, elevated levels of both mRNA and protein expression of *TRIM28* were noted in tumor tissues from patients with liver cancer.^120^ Similarly, examination of tissue samples has demonstrated an escalation in *TRIM28* levels throughout the clinical evolution, rising from approximately 40% in situ invasive breast carcinomas to metastasis in lymph nodes. A significant elevation in *TRIM28* gene expression has been noted across all four intrinsic subtypes of breast cancer (BC), as well as in BC metastases, in comparison with normal tissue.^212^, ^213^ In osteosarcoma, *TRIM28* facilitates the SUMOylation of VPS34 by forming a complex with Plasmacytoma variant translocation‐1 (PVT‐1). This interaction further promotes the ubiquitination and degradation of *TSC2*, consequently enhancing the self‐renewal and stem cell‐like characteristics of osteosarcoma cells.^122^

**FIGURE 2:**
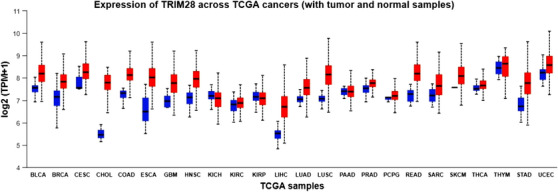
The expression level of *TRIM28* across TCGA cancers. The *TRIM28*’s expression is upregulated in 21 cancers, while in three cancers, there is no significant change. This plot was extracted from UALCAN online database (https://ualcan.path.uab.edu/cgi‐bin/Pan‐cancer.pl?genenam=TRIM28). BLCA, bladder cancer; BRCA, breast cancer; CESC, cervical cancer; CHOL, bile duct cancer; COAD, colon cancer; ESCA, esophageal cancer; GBM, glioblastoma; HNSC, head and neck cancer; KICH, kidney chromophobe; KIRC, kidney clear cell carcinoma; KIRP, kidney papillary cell carcinoma; LIHC, liver cancer; LUAD, lung adenocarcinoma; LUSC, lung squamous cell carcinoma; PAAD, pancreatic cancer; PCPG, pheochromocytoma and paraganglioma; READ, rectal cancer; SARC, sarcoma; SKCM, melanoma; STAD, stomach cancer; THCA, thyroid cancer; THYM, thymoma; UCEC, endometrioid cancer.

Moreover, there exists a positive correlation between the levels of *TRIM28* and the aggressiveness of BC.^198^ Immunohistochemical analysis has unveiled elevated levels of *TRIM28* in a considerable proportion of many cancers, including BC, GC, lung, and so on. This suggests that the upregulation of *TRIM28* is a prevalent characteristic among various epithelial cancers.^212^ To summarize, while numerous studies indicate that increased *TRIM28* levels are correlated with poorer prognoses in certain cancers, conflicting findings have also emerged. For example, in early‐stage lung cancer, heightened *TRIM28* expression is linked to enhanced overall survival, indicating a potential antiproliferative role within tumor cells. Moreover, liver‐specific depletion of *TRIM28* in mice has been shown to elevate male‐predominant hepatic adenoma, indicating a protective role of *TRIM28* against tumorigenesis in liver cells.^194^ Certainly, recent investigations have verified that *TRIM28* acts as a crucial regulator of sexual dimorphism in the liver, tightly managing the expression of numerous genes associated with bile and steroid metabolism.^214^ Hence, *TRIM28* plays a crucial role in nontumor cells by ensuring the maintenance of their unaltered phenotype. The majority of cancers (21 out of 24) have elevated *TRIM28* expression levels, as seen in Figure 2. But only three cancers—KICH, KIRP, and PAAD—show no discernible difference in *TRIM28* expression between normal and cancerous samples. Consequently, it is important to note that *TRIM28* affects many types of cancer. The structure and functions of *TRIM28*, as described, play a significant role in various diseases, particularly in cancer, making its investigation both intriguing and highly relevant. Since studies related to cancer encompass various aspects, the relationship of each aspect with *TRIM28* will be thoroughly examined.

#### 3.2.1 *TRIM28* and cancer cell proliferation

One of the most well‐known tumor suppressor genes is *TP53*, which codes for the *P53* protein.^215^ As an *E3* ubiquitin ligase, murine double minute 2 (*MDM2*) performs a crucial function in the regulation of *P53*. *MDM2* interacts with *P53* and facilitates its ubiquitination, representing the primary mechanism governing the levels of *P53* in cells.^216^ In lung cancer, *RLIM* interacts with *MDM2*, promoting the ubiquitination‐mediated degradation of *MDM2*. Conversely, *TRIM28* binds to *RLIM* and enhances its ubiquitination, leading to decreased levels of *p53* expression. This mechanism ultimately supports tumor cell proliferation and survival.^217^ Besides its involvement in regulating *P53* to influence tumor cell proliferation, *TRIM28* can also stimulate CC cell growth by activating the mTOR signaling pathway.^218^ Likewise, *TRIM28* can promote the growth and migration of endometrial cancer cells by activating the AKT/mTOR signaling pathway. This pathway is known for its involvement in regulating cell growth, survival, and migration.^219^ *TRIM28* can additionally facilitate tumor cell proliferation as it is directly targeted by microRNAs. For example, *miR‐491* is found to be downregulated in glioblastoma multiforme tissues. The reduced expression of *miR‐491* results in elevated levels of *TRIM28*, consequently promoting glioma cell proliferation.^220^ Additionally, *miR‐140‐3p* suppresses the proliferation and migration of BC cells by directly targeting and regulating *TRIM28* expression.^221^

In glioma cells, the suppression of *TRIM28* results in elevated levels of *P21*, leading to cell cycle arrest in the G1 phase.^210^ *UBE2S*, which is found to be overexpressed in hepatocellular carcinoma (HCC), interacts with *TRIM28* in the nucleus, together promoting the ubiquitination of *P27*. This collaborative mechanism aids in facilitating cell cycle progression.^201^ *TRIM28* can also exhibit an antiproliferative effect in early lung cancer by inhibiting the transcriptional activity of the *E2F* family.^194^

#### 3.2.2 The function of *TRIM28* in cancer cell apoptosis

The interaction between *TRIM28* and *MDM2* contributes to the suppression of apoptosis in cancer cells.^197^ The *MAGE* protein, frequently upregulated in various cancer types, acts as a corepressor of *P53* by forming a complex with *TRIM28*.^222^ Reducing *TRIM28* levels in non‐small cell lung cancer leads to decreased expression of the antiapoptotic gene *Bcl*‐2, while simultaneously increasing the expression of the proapoptotic genes *Bax* and *P53*.^218^ *TRIM28* can also negatively regulate the expression of proapoptotic genes, such as *BOK*, at the posttranscriptional level.^223^ Activation of *RIPK3* leads to the phosphorylation of *TRIM28* at *S473*, causing a disruption in *TRIM28*’s ability to bind to chromatin.^224^ In a previous study, the authors have shown that the introduction of *TRIM28* siRNA leads to a reduction in cell proliferation and impedes cell cycle progression in NSCLC cell lines.^218^ The antiapoptotic protein *BCL2A1*, a member of the *BCL‐2* family, is implicated in chemoresistance in certain tumors. Despite its significance, *BCL2A1* has a brief half‐life, largely due to ongoing processing by the ubiquitin–proteasome system, which serves as a crucial tumor‐suppressor mechanism governing *BCL2A1* activity. However, the specific enzymes responsible for modulating *BCL2A1* protein stability remain unknown. Interestingly, endogenous *TRIM28* and *BCL2A1* have been observed to interact with each other at mitochondria. Knockdown of *TRIM28* leads to reduced ubiquitination of *BCL2A1*.^225^

#### 3.2.3 *TRIM28* and epithelial‐to‐mesenchymal transition

The process known as epithelial‐to‐mesenchymal transition (EMT) is characterized by the loss of epithelial traits, notably cell polarity and intercellular connections, coupled with the acquisition of a mesenchymal phenotype.^226^ The initiation of EMT can be prompted by various extracellular stimuli and transcriptional regulators. However, the precise mechanisms through which these disparate signaling pathways coordinate the intricate cellular processes that enable EMT remain incompletely understood.^227^, ^228^ Initially identified during various pivotal phases of embryonic development, EMT has been associated with facilitating carcinoma invasion and metastasis.^229^ Primary features of EMT include diminished levels of cell adhesion molecules, particularly E‐CADHERIN; (ii) EMT entails increased expression of matrix metalloproteinases, facilitating basement membrane degradation; (iii) EMT involves the initiation of the *Rho*/*Cdc42* family small GTPases, crucial for restructuring the cytoskeleton ^230^; and (iv) during EMT, several transcription factors, including *β‐CATENIN*, the *TCF/LEF1* complex, *SNAI1*, *SNAI2*, *SIP‐1*, and *TWIST1*, undergo nuclear translocation.^231^ Moreover, the presence of specific EMT inducers has been identified in various human cancer specimens, including breast carcinomas.^227^, ^229^ A recent breakthrough has revealed a new central controller of EMT, consisting of a protein–DNA compound featuring TRIM*28* protein, *CBF‐A*, and the *FTS‐1* element. This compound holds significant importance in managing the expression of *FSP1* in fibroblasts.^192^

The *CBF‐A* and TRIM*28* proteins possess the capability to identify and attach to the *FTS‐1* sites within genomic DNA, consequently regulating the expression of a wide range of EMT‐responsive genes.^232^ The presence of these proteins at the *FTS‐1* site within chromatin of epithelial cells undergoing transition to fibroblasts is associated with the activation of the EMT proteome.^192^ Recent studies suggest that *TRIM28* has the ability to facilitate the infiltration of cancer cells by regulating EMT.^16^ Likewise, it has been noted that heightened levels of *TRIM28* prompt EMT in pancreatic cancer cells, both in laboratory settings and within living organisms.^233^ Furthermore, studies have demonstrated that *TRIM28* is implicated in TGF‐β‐triggered EMT in non‐small cell lung cancer cells.^234^ A recent report has unveiled that *TRIM28* fosters BC metastasis by stabilizing the *TWIST1* protein, acknowledged as a transcription factor pivotal in serving as a regulator of EMT.^198^

Both overexpression and depletion of *TRIM28* resulted in changes in *TWIST1* protein levels, with upregulation and downregulation, respectively, without affecting *TWIST1* mRNA levels. Knockdown of *TRIM28* caused *TWIST1* to be downregulated, accompanied by increased *E‐CADHERIN* expression and decreased *N‐CADHERIN* expression.^198^ Importantly, EMT plays a pivotal role in obtaining and maintaining stem cell‐like traits, capable of imparting stem cell features to both differentiated normal and cancerous cells.^235^ Moreover, cancer stem cells (CSCs) often exhibit characteristics associated with EMT. The bidirectional interaction between EMT and CSCs carries substantial implications for tumor progression. Considering the known role of TRIM*28* protein in governing EMT, there arises the possibility of its involvement in sustaining CSCs. Consequently, *TRIM28* emerges as a promising candidate as an epigenetic modulator that promotes cellular metastasis. Therefore, it could represent a promising therapeutic target for addressing cancer, especially when used in conjunction with conventional treatment modalities.^196^

#### 3.2.4 *TRIM28* and CSC attributes

ESCs have been widely employed as a model to explore the molecular mechanisms underlying self‐renewal and pluripotency.^236^ The role of TRIM*28* protein in mouse embryonic development was first clarified in 2000.^237^ *TRIM28* plays a critical role in preserving cells in their inherent “state of cell differentiation,” thereby promoting stem cell maintenance while also impeding the reprogramming of somatic cells.^238^ Observations revealed that the reduction of *TRIM28* led to a notable decrease in the mRNA expression levels of *Oct‐4*, *Sox2*, and Nanog, subsequently triggering the differentiation of ESCs towards the primitive ectoderm lineage.^238^ Alongside other pluripotency markers like *Cnot3*, *Zfx*, and *c‐Myc*, *TRIM28* simultaneously binds to numerous potential gene promoters.^238^ Moreover, investigations have demonstrated that phosphorylated TRIM*28* protein at *Ser824* interacts with the pluripotency‐specific transcription factor *Oct‐4* on the promoters of genes associated with pluripotency. As a result, this mechanism contributes to the efficient regulation of pluripotent ESCs in a manner dependent on phosphorylation.^239^ Furthermore, it has been reported that *TRIM28* exhibits contrasting effects on differentiation‐inducible markers in mouse ESCs, in contrast to its role in inducing the expression of pluripotency markers.^240^ *TRIM28* has been identified as an epigenetic barrier to induced pluripotent stem (iPS) cell reprogramming.^241^ Interestingly, it has been documented that while depletion of *TRIM28* relaxes chromatin and facilitates the reversion to a differentiated state, iPS cells generated with reduced *TRIM28* expression swiftly lose their self‐renewal potential and undergo spontaneous differentiation. This underscores the crucial role of *TRIM28* in sustaining stable iPS cells.^242^ Recently, the crucial role of *TRIM28* in sustaining the population of BC stem cells has been documented.^243^

CSCs represent a small subset of cells within tumors characterized by their possession of stem cell‐like attributes, such as self‐renewal capacity, pluripotency, heightened tumorigenic potential, and resistance to therapy.^244^ The suppression of *TRIM28* has been demonstrated in the highly aggressive, undifferentiated cells of the triple‐negative BC MDA‐MB‐231 cell line.^212^ Additionally, the depletion of *TRIM28* led to a confirmed decrease in the population of CSCs in MDA‐MB‐231 xenografts, as validated by in vivo limiting dilution assays. Furthermore, it was observed that *TRIM28*, in partnership with *EZH2*, a component of the *PRC2* complex, collectively regulates a subset of genes related to stem cell maintenance and associated with unfavorable survival outcomes in BC patients.^243^ The depletion of *TRIM28* in the MCF7 BC cell line in vitro resulted in a notable decrease in the formation of CD44+ CD24−/low mammospheres, which was accompanied by a reduction in the expression of genes associated with stem cells. These findings underscore the significant role of *TRIM28* in activating gene expression that sustains the enrichment and maintenance of mammary stem cells, thereby emphasizing its involvement in promoting cancer progression.^238^, ^243^ *CD133* serves as a well‐studied marker for the most malignant tumor cell population, known as CSCs. However, the precise function of this glycoprotein and its involvement in cellular regulatory pathways remain inadequately understood. Notably, among these pathways, the *TRIM28* transcription factor holds particular significance. Experimental evidence, particularly in Caco2 cell line clones, confirms the pivotal role of *TRIM28* in modulating *CD133* expression. Specifically, knockout of the *TRIM28* gene resulted in the downregulation of *CD133* expression. These findings underscore the significant contribution of the *TRIM28* transcription factor in regulating the heterogeneity of cancer cells associated with *CD133*.^245^

#### 3.2.5 *TRIM28* and cancer cell metabolism

Cancer cells employ diverse strategies, such as increased glycolytic activity, modulation of redox signaling, and regulation of autophagy, to evade cell death and counteract nutrient deprivation.^246^ Recent studies have shown that autophagy is mechanistically linked to the preservation of CSCs, allowing them to survive drug toxicity.^247^, ^248^ Remarkably, the TRIM*28* protein is involved in regulating autophagy at multiple levels.^190^, ^191^, ^200^ There have been reports indicating a notable involvement of the TRIM*28* protein in orchestrating phagophore formation.^200^ The initiation and nucleation of the phagophore rely on the activity of AMP‐AMPK and *ULK1/2* complex, which is regulated by the mTORC1.^249^ VPS34 recruits other autophagy regulatory proteins essential for phagophore and autophagic vesicle (AV) formation.^250^ In conjunction with its binding partner, *BECLIN1*, *VPS34* forms a complex that is additionally associated with the TRIM*28* protein. Through its role as a *SUMO* E3 ligase via its *PHD* domain, *TRIM28* facilitates the SUMOylation of *VPS34* at *Lys840*, consequently augmenting its activity and promoting autophagosome formation.^200^ Additionally, *TRIM28* is implicated in the regulation of mitophagy, a process that involves the selective degradation of mitochondria by autophagy.^251^ This interaction leads to reduced AMPK signaling, increased mTORC signaling, and subsequent inhibition of autophagy.^247^, ^248^ Moreover, it has been shown that the assembly of *MAGE–TRIM28* ubiquitin ligase complexes enhances the Warburg effect and facilitates the progression of HCC by directing the degradation of *FBP1*, a key enzyme in gluconeogenesis.^195^

#### 3.2.6 *TRIM28* and silencing of TEs

Genetic instability is a key characteristic linked to the development and advancement of cancer. TEs are among the factors contributing to this instability, as they can cause various types of cancer by inserting mutations into specific genes that are crucial for either suppressing or promoting malignant transformation.^252^ Increased levels of *TRIM28* have been noted to enhance the utilization of glucose and the generation of lactate by facilitating the degradation of *FBP1* in HCC. This action promotes the proliferation of cancer cells both in laboratory settings and in a murine model, indicating a significant role for *TRIM28* in promoting tumor growth. These discoveries underscore the protumorigenic properties of *TRIM28*. Clearly, the interplay between *TRIM28* and the metabolic status of cancer cells is intricate and requires additional investigation.^253^

In human cells, TEs encompass both DNA transposons and RNA transposons, commonly referred to as retroelements. The activity of RNA transposons is meticulously regulated in ESCs through a precisely orchestrated epigenetic mechanism involving *KRAB*‐*ZNF* repressors in conjunction with their cofactor, *TRIM28*.^254^, ^255^ Ten years ago, *TRIM28* was identified as a component of the repressor binding site‐binding complex, which plays a role in epigenetically silencing retroviruses in both ESCs and embryonic carcinoma cells.^254^, ^256^ Reducing the expression of *TRIM28* resulted in the depletion of the repressive chromatin modification H3K9me3 specifically at 5′UTR of IAP genomes. This alteration led to the heightened expression of *IAPs*.^254^ The regulation imposed by *TRIM28* on retroelement expression is vital for maintaining the transcriptional dynamics during early embryo development. However, the absence of *TRIM28* in mouse embryonic fibroblast cells did not result in increased retrotransposon expression.^255^ This observation suggests that the silencing of retrotransposons plays a minimal role in differentiated cells.^255^ A recent discovery highlighted the involvement of *TRIM28* in the suppression of LINE‐1 in mouse fibroblasts. *TRIM28* coordinates the compaction of these TEs into transcriptionally inactive heterochromatin, a process instigated by mono‐ADP ribosylation facilitated by the *SIRT6* protein.^257^ This modification is crucial for recruiting HP1α and other silencing factors to the target locus. However, the mechanism by which *TRIM28* is directed to specific chromatin sites where the L1 retroelement is located remained unclear.^258^ Similar findings were also observed in human ESCs.^259^

However, it remains uncertain whether cancer cells operate under similar mechanisms. Unlike normal cells, many human cancers and cancer‐derived cell lines exhibit variable, yet generally elevated levels of endogenous full‐length L1 mRNA expression, even in the presence of increased *TRIM28* activity.^260^ A novel *URI*–*PP2A*–*TRIM28* complex has been reported to play a substantial role in regulating L1 expression in prostate cancer cells, representing a recent advancement in our understanding of *L1* regulation.^199^ *URI*, identified as a transcriptional repressor interacting with RNA polymerase II, has been shown to associate with and facilitate the dephosphorylation of DNA‐bound *TRIM28*–*Ser824* by recruiting the *PP2A* phosphatase. This process leads to the transcriptional suppression of the *L1* retroelement.^261^ Researchers observed a significant elevation in *L1* mRNA expression subsequent to *TRIM28*–*Ser824* phosphorylation, accompanied by chromatin decondensation. This phosphorylation was triggered by URI depletion, leading to the prevention of *PP2A* recruitment to *TRIM28* phospho‐*Ser824*.^199^

#### 3.2.7 Shared pathway in cancers and *TRIM28*

**FIGURE 3:**
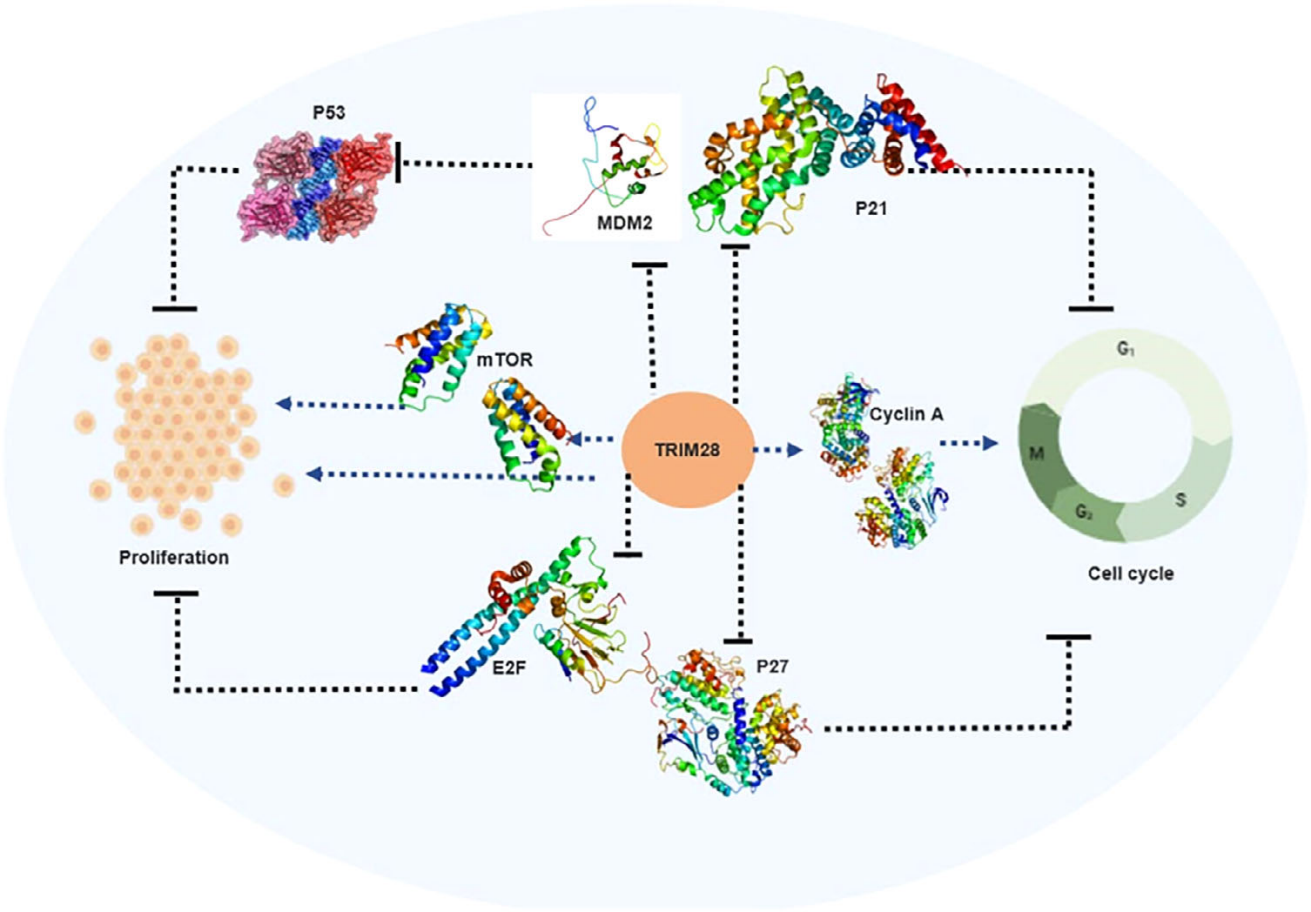
*TRIM28* signaling pathway and its corresponding effectors. This figure depicts the key proteins and pathways related to *TRIM28* that are involved in cell proliferation, survival, genomic stability, and various cancer types. This figure was created using BioRender and Microsoft Publisher 2016.

*TRIM28* exhibits a multifaceted role in cancer pathogenesis by regulating diverse signaling pathways implicated in cell proliferation, survival, and genomic stability. Unraveling the precise mechanisms by which *TRIM28* influences different cancer types could offer valuable insights for the design of targeted therapeutic approaches (Table 4 and Figure 3). The development of targeted therapeutic approaches may benefit from a better understanding of the precise mechanisms by which *TRIM28* influences various cancer types. This might be taking advantage of its regulatory networks to make cancer cells more susceptible to current therapies or creating brand‐new inhibitors that precisely block its carcinogenic properties. Moreover, *TRIM28*’s capacity to engage with the immune system, especially via its function in immune regulation and inflammation, emphasizes the possibility of using it as a therapeutic target in the tumor microenvironment in addition to cancer. Gaining insight into the entire range of *TRIM28*’s roles in cancer biology may help create more specialized and successful cancer treatments.

**TABLE 4:** The association of *TRIM28* with various cancers through signaling pathways.

Type of cancer	Signaling pathway	References
CC	mTOR	^218^
BC	EMT	^198^
GC	TBK1‐IRF1 and TBK1‐mTOR	^262^
Endometrial cancer	AKT/mTOR	^219^
Ovarian carcinoma	Wnt/β‐catenin/Shh	^263^, ^264^
Thyroid cancer	Wnt/β‐catenin	^265^
Colorectal cancer	EMT	^266^
Bladder cancer	mTOR	^267^
Glioblastoma	Autophagy	^268^
B‐cell non‐Hodgkin lymphoma cells	p21/ promoting the cell cycle progression	^269^
Lung cancer	p53	^217^
Non‐small cell lung cancer	p53	^218^
Prostate cancer	Androgen receptor	^270^

Abbreviations: AKT, serine/threonine kinase; Shh, sonic hedgehog pathway; EMT, epithelial–mesenchymal transition; IRF1, interferon regulatory factor 1; mTOR, mammalian target of rapamycin; TBK1, TANK‐binding kinase 1; Wnt, Wingless‐related integration site; β‐catenin, beta‐catenin.

#### 3.2.8 The involvement of *TRIM28* in immune modulation

Targeting *TRIM28* offers a promising strategy for therapeutic intervention to enhance radiation‐induced antitumor immune responses.^271^ The *SETDB1*–*TRIM28* complex acts as a major inhibitor of antitumor immunity.^272^ Melanoma exhibiting high level of *TRIM28* expression demonstrated a significant downregulation of members of the IRF transcription factor family, including *IRF1*, *IRF2*, *IRF5*, and *IRF8*, which correlated with diminished IFN signaling.^273^ Moreover, *TRIM28* acts as a specific SUMO E3 ligase for *IRF7*. It also interacts with the N‐terminus of *IRF5*, thereby inhibiting its function in inflammatory macrophages.^274^ *TRIM28* associates with the transcriptional regulator *FOXP3* in human Tregs.^275^, ^276^

Notably, *TRIM28* knockdown has been associated with increased responsiveness to *anti‐PD‐1* therapy in immunocompetent mice, evidenced by higher levels of CD8+ T tumor‐infiltrating lymphocytes and decreased numbers of myeloid‐derived suppressor cells.^277^

The *SETDB1*–*TRIM28* complex has been found to exhibit a negative correlation with the infiltration of effector CD8+ T cells. Inhibiting the *SETDB1*–*TRIM28* complex has been shown to simultaneously upregulate PD‐L1 expression and activate the cyclic GMP–AMP synthase cGAS–STING innate immune response pathway, thereby increasing the infiltration of CD8+ T cells. Mechanistically, inhibition of *SETDB1*–*TRIM28* results in the formation of micronuclei in the cytoplasm, which is known to activate the cGAS–STING pathway.^272^

#### 3.2.9 *TRIM28* and autophagy

Autophagy has a multifaceted role in cancer, exhibiting both protumorigenic and antitumorigenic effects that vary with the stage and context of the disease. TRIM proteins, as regulators of autophagy, play a significant role in this dual nature, acting either as proto‐oncogenes or antioncogenes. For instance, the *MAGEA3* or 6 interacts with *TRIM28* to ubiquitinate and degrade *PRKAA2*, which leads to autophagy inhibition through the mTOR pathway and subsequently promotes tumorigenesis. In the absence of *MAGEA3* or 6, *TRIM28* instead promotes autophagy by SUMOylating *PIK3C3*, which facilitates the formation of the *BECN1* complex. Additionally, *TRIM28* is implicated in the pathogenesis of glioblastoma by inducing autophagy.^278^, ^279^ *TRIM28* functions as both a proautophagy and antiautophagy factor under different circumstances, reflecting a dichotomy resulting from the coordinated actions of multiple factors. For instance, *TRIM28* can inhibit autophagy by associating with *MAGEA3* or 6 to ubiquitinate and degrade *PRKAA2*, thereby activating the mTOR pathway and promoting tumorigenesis. Conversely, in the absence of *MAGEA3* or 6, *TRIM28* can promote autophagy through the SUMOylation of *PIK3C3*, which enhances *BECN1* complex formation. This dual functionality underscores the complex regulatory role of *TRIM28* in autophagy, influenced by its interactions with various molecular partners.^268^ It has been reported that *TRIM28* is involved in the autophagy process. Evidence indicates that *TRIM28* activates autophagy and enhances cell proliferation in glioma.^268^ To put it differently, autophagy assumes different functions at distinct phases of tumor advancement. Specifically, it typically impedes tumor advancement during the initial phases of cancer progression, whereas it fosters tumor expansion in later stages.^200^ *TRIM28* is indispensable for the synthesis of inosine phosphate and the generation of AVs in the process of autophagy.^200^ *TRIM28* also participates in the control of mitophagy, which involves the targeted breakdown of mitochondria via autophagy.^251^ In gliomas, there is a notable increase in the expression levels of *TRIM28* and autophagy as the tumor grade advances. Suppression of *TRIM28* can impede autophagy in glioblastoma cells.^268^ *TRIM28* has the capability to degrade AMPK, a cellular energy sensor and regulator, through ubiquitination pathways. This degradation leads to a substantial decrease in autophagy, changes in cellular metabolism, and activation of mTOR signaling.^190^ The *MAGE–TRIM28* axis also influences the Warburg and the advancement of HCC by targeting *FBP1* and promoting its degradation. This suggests that the *MAGE*–*TRIM28* axis governs the metabolic reprogramming of cancer cells by regulating the degradation of various metabolic regulators.^195^ *TRIM28* appears to play a crucial role in regulating tumor cell apoptosis, necroptosis, and autophagy. Nonetheless, its association with pyroptosis and ferroptosis remains unexplored. Investigating whether *TRIM28* is implicated in these two forms of cell death is essential for a comprehensive understanding of its role in tumor biology.

#### 3.2.10 *TRIM28* and necroptosis

Necroptosis is a type of cell death that induces inflammation and, while it shares some morphological similarities with necrosis, it is fundamentally different. Unlike necrosis, necroptosis is a regulated process akin to apoptosis, involving specific death signaling pathways that lead to controlled cell death. The TNF (tumor necrosis factor)/TNFR (TNF receptor) signaling pathway is a well‐established trigger for necroptosis. When TNF binds to TNFR1, it activates downstream signaling pathways. Under certain conditions, such as the inhibition or deficiency of caspase‐8, TNFR1 signaling can initiate the necroptosis pathway, resulting in necroptotic cell death. This pathway has been thoroughly investigated and serves as a critical model for elucidating the mechanisms and regulation of necroptosis.^280^, ^281^ *RIPK1*, an upstream regulator, is indeed considered a key signaling node in the TNF signal transduction pathway. Depending on the cellular environment and the presence of other signaling molecules, *RIPK1* can determine whether TNF stimulation results in cell survival, apoptosis, or necroptosis. RIPK1's role is intricately regulated and essential for maintaining cellular homeostasis and responding to stress signals. When apoptosis is blocked, such as due to inhibited or deficient caspase‐8 activity, *RIPK1* forms a complex with *RIPK3* known as the necrosome. This necrosome complex is crucial for mediating necroptotic cell death, acting downstream of various death receptor signaling pathways, including TNF/TNFR1. The necrosome activates downstream effectors that drive necroptosis, a controlled form of necrotic cell death.^282^ The necrosome phosphorylates the activation loop of *MLKL* (mixed‐lineage kinase domain‐like protein), which is the essential final effector in the necroptotic pathway. Once phosphorylated, *MLKL* translocates to the plasma membrane, where it compromises membrane integrity. This disruption leads to the release of intracellular contents and triggers inflammation, hallmark features of necroptotic cell death.^283^, ^284^, ^285^ Activation of *MAPK14* (mitogen‐activated protein kinase 14), driven by oligomerized *MLKL* (mixed lineage kinase domain‐like pseudokinase), leads to the phosphorylation of *TRIM28* at *S473*. This phosphorylation promotes inflammation in the later stages of necroptosis, revealing how RIPK1 kinase activation influences inflammatory responses. Necroptosis enhances tumor immunogenicity and is being explored as a target for cancer immunotherapy due to its ability to induce inflammatory protein synthesis and stimulate antitumor immune responses. *TRIM28*, a corepressor in necroptosis, is phosphorylated by *RIPK3*, reducing its chromatin‐binding ability and thereby increasing the activation of *NFKB* and other transcription factors.^224^

#### 3.2.11 The potential of *TRIM28* as a target for cancer therapy

Cytotoxic chemotherapeutic agents and targeted therapies are pivotal in clinical oncology. Nevertheless, the development of drug resistance in tumor cells significantly compromises the efficacy of these anticancer treatments.^286^ Reducing *TRIM28* expression significantly increases the vulnerability of lung cancer cells to 5‐fluorouracil.^287^ Bortezomib (BTZ), a selective inhibitor of proteasomes, shows potential in HCC therapy. Nonetheless, drug resistance poses a considerable challenge to its effectiveness. Remarkably, *TRIM28* diminishes BTZ sensitivity in HCC cells by enhancing proteasome expression.^288^ The expression of *TRIM28* is notably elevated in castration‐resistant prostate cancers. *TRIM28* overexpression amplifies androgen receptor signaling, representing a pivotal mechanism in antiandrogen deprivation therapy for prostate cancer.^270^ *E2F1* is a frequently targeted molecule in the development of anticancer chemotherapeutics.^289^ This phenomenon may be linked to the expression of *TRIM28*, which recruits *HDAC1* to deacetylate *E2F1*, thereby suppressing its activity. Additionally, the downregulation of *TRIM28* enhances cell death induced by etoposide.^33^ Following DNA damage, phosphorylation of *TRIM28* at *Ser473* boosts its interaction with E2F1. Utilizing combination chemotherapy involving etoposide along with a suitable *TRIM28* S473p‐blocking peptide might aid in inducing tumor cell death. Additionally, the *P53* pathway represents a significant focus in cancer drug development.^290^

In cells where *TRIM28* is depleted, exposure to actinomycin D results in the activation of *P53*, suggesting that *TRIM28* could serve as a target for *P53* reactivation in cancer cells. Coadministration of actinomycin D with compounds that disrupt the interaction between *TRIM28* and MDM2 or decrease *TRIM28* levels can effectively inhibit MDM2 function, thereby significantly activating *P53*.^196^, ^291^ The peroxide‐induced activation of *P38* MAPK promotes the phosphorylation of *TRIM28* at *S473*, resulting in the activation of *TRIM28*. This activated state effectively assists DNA repair mechanisms, thereby aiding tumor cells in surviving exposure to exogenous ROS.^292^ Hence, inhibiting *TRIM28* may render tumor cells more susceptible to chemotherapy.

**FIGURE 4:**
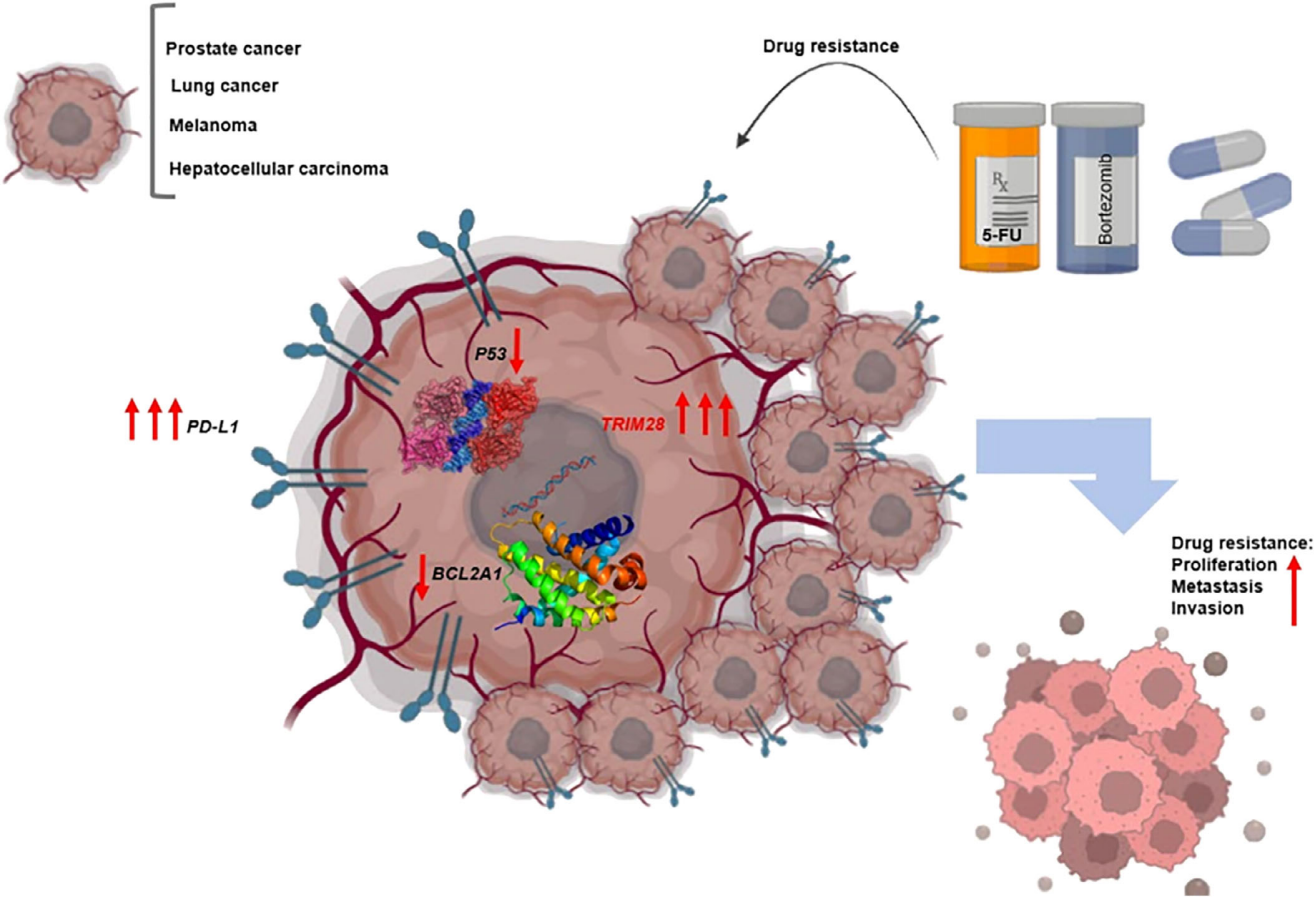
*TRIM28* in cancer therapy potential. The list of some cancers linked to TRIM28 treatment is visualized in this figure, emphasizing *TRIM28*’s function in therapeutic interventions, its potential as a target for therapeutic approaches, and the clinical results. This figure was created using BioRender and Microsoft Publisher 2016.

A wide range of nanobodies has been utilized in clinical trials aimed at treating various diseases across the spectrum.^293^ The specific anti‐*TRIM28* nanobody, *NB237*, has been demonstrated to significantly inhibit the invasion and metastasis of both glioblastoma cells and glioblastoma stem cells within the zebrafish brain.^294^ Research conducted in cardiomyocytes has demonstrated that ZFG protects against hypoxia/reoxygenation‐induced apoptosis by decreasing the expression of *TRIM28*.^295^ There is an urgent need for the development of small molecule inhibitors targeting *TRIM28*, which could potentially serve as adjuncts to tumor therapy.^267^ *PD‐L1* plays a crucial role in facilitating tumor‐induced immunosuppression,^296^ and verteporfin has emerged as a significant small molecule inhibitor of *PD‐L1*. Studies suggest that verteporfin can inhibit both basal and IFN‐induced *PD‐L1* expression, both in laboratory settings and within living organisms, by targeting Golgi‐associated autophagy and disrupting the *STAT1*–*IRF1*–*TRIM28* signaling cascade.^297^ Collectively, *TRIM28* appears to contribute to drug resistance in certain cancers and is a significant factor rendering certain anticancer drug targets unresponsive to treatment. Advancing tumor treatment goals may involve the development of drugs or small molecules specifically targeting *TRIM28*. Table 5 provides a list of some cancers related to *TRIM28* treatment. This aspect remains a gap in current *TRIM28* research efforts (Figure 4).

**TABLE 5:** Evidence related to *TRIM28* in treatment.

Cancer	Clinical evidence	References
Lung	Reduction of *TRIM28* expression enhances the sensitivity to *5‐FU*, etoposide, and cisplatin.	^298^
	*TRIM28* can upregulate *miR‐125b‐5p* and plays a significant role in *DDP* resistance in patients.	^299^
	Knockdown of *TRIM28* triggers cell apoptosis by promoting *E2F1* inactivation and decreasing cellular sensitivity to etoposide treatment.	^300^
HCC	Reduces sensitivity to *BTZ* by upregulating proteasome expression.	^288^
	*TRIM28* can increase sensitivity to oxaliplatin therapy by facilitating the ubiquitin‐mediated degradation of *HMGB1*.	^301^
	Elevated *TRIM28* expression contributes to resistance against *TKI* therapy.	^15^
Pancreatic	Overexpression of *TRIM28* results in enhanced degradation of *FBP1*, which subsequently prevents the degradation of *c‐Myc*, thereby increasing cellular resistance to bromodomain inhibitor.	^302^
Glioma	ATM inhibitor (ATMi) drugs enhance cellular sensitivity to radiotherapy by blocking *TRIM28* phosphorylation.	^303^
Various cancers	Verteporfin may inhibit *PD‐L1* expression by disrupting the interaction between *TRIM28* and *IRF1*.	^297^

Abbreviations: 5‐FU, 5‐fluorouracil; BTZ, bortezomib; c‐Myc, cellular myelocytomatosis oncogene; DDP, cisplatin; *FBP1*, fructose‐bisphosphatase 1; HMGB1, high mobility group box 1; IRF1, interferon regulatory factor 1; PD‐L1, programmed cell death 1 ligands; *TKI*, tyrosine kinase inhibitors.

### 3.3 *TRIM28* and infertility

Haploinsufficiency, a process where a loss‐of‐function mutation results in only a single functional copy of a gene, leading to an inadequate production of the gene's product, is a significant contributor to various developmental disorders, including male infertility.^304^ Phenotypes associated with haploinsufficiency usually involve critical genes that encode transcription factors or are involved in developmental pathways.^305^, ^306^, ^307^, ^308^ It is not surprising that numerous epigenetic factors involved in development, such as *NSD1*, *SUZ12*, *TRIM28*, and others, have been found to exhibit haploinsufficiency phenotypes.^309^ *TRIM28* serves as a crucial mediator of epigenetic modifications and is essential for development, as demonstrated by the embryonic lethality observed in both zygotic and maternal *TRIM28* knockout mice, and the rapid mortality in adult mice following systemic *TRIM28* deletion (Table 6). Functionally, *TRIM28* is involved in a wide range of biological processes, including cell differentiation, the DNA damage response, suppression of retroviral elements, and the epigenetic inheritance from germline to soma.^7^, ^237^, ^310^, ^311^, ^312^ *TRIM28* haploinsufficiency has been demonstrated to induce a bistable obesity phenotype in mice, which is linked to the dysregulation of nonclassically imprinted genes.^313^ One report reveals a new aspect of *TRIM*28 haploinsufficiency: a highly penetrant infertility phenotype observed in male mice heterozygous for *TRIM28* (*TRIM28Het*). Currently, *TRIM28* is known to be expressed in spermatocytes and round spermatids and is essential for normal spermatogenesis. However, the underlying mechanism of testicular degeneration following *TRIM28* deletion remains unclear, with a potentially paracrine, noncell‐autonomous mechanism being suggested.^314^, ^315^ Premature ovarian insufficiency (POI) is a clinical condition characterized by ovarian dysfunction and abnormal alterations in hormone levels, such as follicle‐stimulating hormone and estradiol (E2). Research has shown that POI is associated with the cellular senescence of ovarian granulosa cells, with *TRIM28* playing a pivotal role in regulating oxidative stress (OS)‐induced cellular senescence in these cells. Mechanistically, OS decreases TRIM28 protein levels in KGN cells, leading to increased expression of autophagy markers *ATG5* and *LC3B‐II*, alongside the downregulation of *P62*.^316^

**TABLE 6:** Effects of *TRIM28* on diseases in animal studies.

Description	Animal	Disorder	References
*TRIM28* likely functions as an E3 SUMO ligase, affecting the stability and subcellular localization of α‐Syn and tau via SUMOylation.	Mice	Parkinson's disease and Alzheimer's disease	^317^
*TRIM28*, a crucial epigenetic regulator, undergoes progressive testicular degeneration.	Mice	Infertility	^315^
*TRIM28* haploinsufficiency results in a stochastic bi‐stable phenotype, characterized by the development of obesity or an alternative, nonobese state, also known as polyphenism.	Mice	Epigenetic obesity	^313^
Ectopic expression of *TRIM28* promoted tumor growth, elevated PD‐L1 expression, and inhibited T cell activation.	Mice	GC	^262^
*TRIM*28 protein modulates the tumor growth of the CSC population.	Mice	BC	^212^
*TRIM*28 was highly enriched in the core of the tumor and correlated with the expression of stem cell‐related genes.	Zebrafish	Glioblastoma	^294^
The deletion of *TRIM28* in skeletal muscle of mice, either during development (MCK–cre) or after development (ACTA1–cre–ERT2), does not confer protection against high‐fat diet (HFD)‐induced obesity or glucose intolerance	Mice	Diabetes	^318^

Abbreviations: ACTA1, actin alpha 1; ERT2, estrogen receptor T2; HFD, high‐fat diet; MCK, muscle creatine kinase.

### 3.4 *TRIM28* and obesity

The effective storage of lipids in white adipose tissue (WAT) is essential for maintaining systemic energy homeostasis. Numerous genes have been implicated in the regulation of WAT lipid metabolism, including *TRIM28*, which has traditionally been associated with influencing adiposity through epigenetic mechanisms during embryonic development. However, the present study reveals that mice with adipocyte‐specific *TRIM28* deletion also exhibit obesity, similar to the phenotype seen in models with global *TRIM28* deletion, suggesting a role for *TRIM28* beyond its developmental functions (Table 6). Notably, the obesity phenotype was significantly more evident in female mice, suggesting that *TRIM28* acts as a sex‐specific regulator of obesity. Mechanistically, this phenotype is associated with changes in lipolysis and triglyceride metabolism, which can be attributed in part to the downregulation of *Klf14*—a gene recognized for its role in modulating adipocyte size and body composition in a sex‐specific context. These findings position *TRIM28* as a crucial sex‐specific regulator of adiposity after developmental and the function of WAT.^319^

### 3.5 *TRIM28* and diabetes

Diabetes mellitus (T2DM), characterized by insulin resistance and mitochondrial dysfunction in skeletal muscle, is a major cause of mortality in developed countries. There is considerable interest in targeting mitochondrial health, including autophagy pathways, as potential strategies for preventing or treating T2DM. In one study, two distinct muscle‐specific *TRIM28* knockout models (MCK–cre and ACTA1–cre–ERT2) were examined under both chow and high‐fat diet (HFD) conditions (Table 6). Despite muscle‐specific *TRIM28* deletion leading to alterations in markers of mitochondrial activity and autophagy in skeletal muscle, most metabolic parameters were largely unaffected. In particular, the absence of *TRIM28* in skeletal muscle, whether during development (MCK–cre) or postdevelopment (ACTA1–cre–ERT2), did not protect against HFD‐induced obesity or glucose intolerance.^318^ These findings are consistent with prior research on autophagy and mitochondrial function in other cell types, indicating that the role of *TRIM28* in regulating mitochondrial function requires further investigation for a more comprehensive understanding.

### 3.6 *TRIM28* and COVID‐19

Severe acute respiratory syndrome coronavirus 2 (SARS‐CoV‐2), the causative agent of coronavirus disease 2019 (COVID‐19), utilizes angiotensin‐converting enzyme 2 (*ACE2*) as a receptor to enter human cells.^320^ The expression level of *ACE2* may impact both the susceptibility to and severity of COVID‐19, underscoring the importance of understanding the regulatory mechanisms that control *ACE2* expression. *TRIM28*, known for its roles in antiviral defense, maintaining endogenous retrovirus latency, and modulating immune responses, has recently been identified as coexpressed with the SARS‐CoV‐2 receptor in type II pneumocytes. However, its role in regulating *ACE2* expression and facilitating SARS‐CoV‐2 cell entry into cells remains unclear. The study demonstrated that *TRIM28* knockdown resulted in increased *ACE2* expression and enhanced pseudotyped SARS‐CoV‐2 entry into A549 cells and primary pulmonary alveolar epithelial cells.^321^

### 3.7 *TRIM28* and other viral diseases

*TRIM28* plays a major role in cellular defense against viral infections. The immune system, cellular homeostasis, and the body's antiviral responses all depend on it. As an adaptable regulator, *TRIM28* affects the host immune system's modulation as well as the direct suppression of viral gene expression.^322^ Beyond only reacting to active infections, *TRIM28* plays a critical role in keeping some viruses in their latent state, which stops reactivation and the subsequent spread of disease. *TRIM28*’s significance in maintaining the equilibrium of the host's defense mechanisms is further demonstrated by its role in immune regulation. It adjusts inflammatory responses by regulating not only the expression of IFN‐stimulated genes but also important signaling pathways like NF‐ĸB. This regulatory ability guarantees a sufficiently strong immune response. Next, we discuss the role of *TRIM28* in other viral diseases.

The HIV‐1 *Tat* protein, which is necessary for the transcription of the HIV‐1 genome, interacts with *TRIM28*. This interaction promotes viral replication. Moreover, *TRIM28* can affect latency and reactivation phases of the HIV life cycle. Histone deacetylases (HDACs) and histone methyltransferases are among the chromatin‐modifying enzymes that *TRIM28* enlists as transcriptional repressors. By inhibiting the expression of viral genes, these enzymes help to form repressive chromatin structures surrounding the proviral DNA, which keeps HIV latent and transcriptionally inactive.^323^ As a result, targeting the *TRIM28*–*Tat* interaction could offer a novel approach to modulating the transcriptional activity of HIV‐1, providing a new avenue for antiviral drug development.

*TRIM28* plays a significant role in the regulation of human papillomavirus (HPV) transcription. It can function as an *E2F* corepressor, which is a crucial transcription factor that HPV targets. This interaction may influence the viral life cycle, including viral replication and persistence, as well as the progression of HPV‐related cancers. HPV infection is strongly associated with the development of various cancers, particularly CC.^324^ The virus expresses oncogenes, such as *E6* and *E7*, which interfere with tumor suppressor proteins like *p53* and retinoblastoma, driving the progression toward malignancy.^324^ *TRIM28*, by regulating transcription factors and chromatin structure, may influence the expression of these oncogenes. Its role in forming repressive chromatin structures could potentially suppress the expression of viral genes involved in oncogenesis, thereby impacting cancer progression.^325^ Hence, understanding *TRIM28*’s role in these processes could provide insights into novel therapeutic strategies for treating HPV‐associated diseases.

Hepatitis B virus X protein (*HBX*) is a multifunctional viral protein and plays an important role in HBV replication and pathogenesis. Through its role as a transcriptional repressor, *TRIM28* can affect the transcriptional regulation of viral host genes and HBV replication and interacts with the *HBX* protein. This interaction has a significant impact on various aspects of the HBV life cycle, including viral replication, transcription, and persistence.^326^ The interaction between *TRIM28* and *HBX* is particularly important in the context of HBV‐induced liver diseases, including chronic hepatitis, liver cirrhosis, and HCC. Therefore, the imbalance between this interaction may affect the development of these diseases. In addition, *TRIM28* is involved in the regulation of immune‐related genes, and its interaction with HBX may help the virus to evade the host's immune system, leading to chronic infection and an increased risk of liver cancer.^326^ Therefore, molecular understanding of this interaction can reveal new targets for therapeutic intervention and provide potential strategies to control HBV replication, reduce liver disease progression, and prevent HCC development.

*TRIM28* plays an important role in regulating various stages of hepatitis C virus (HCV) replication and assembly. It can modulate the efficiency of viral RNA synthesis and help the virus to infect cells. In addition, chronic HCV infection is one of the main causes of liver diseases, where *TRIM28* modulates viral replication and particle aggregation, may contribute to the chronicity of infection at these critical stages.^327^ Therefore, *TRIM28* can be considered as a potential therapy to manage HCV infection and prevent its progression to chronic liver diseases.

Viral infections are intricately linked to the development of tumors. Herpesviruses possess the capability to endure within infected hosts for extended periods without generating viral particles.^274^ *TRIM28* assumes a significant role in tumors associated with herpesviruses, including Epstein–Barr virus (EBV), Kaposi's sarcoma‐associated herpesvirus (KSHV), and human cytomegalovirus.^328^, ^329^, ^330^ *TRIM28* has the capability to sustain the latent phase of EBV and KSHV by binding to the promoters of viral genes.^328^ *TRIM28* becomes a target of latent membrane protein‐1 (*LMP1*)‐induced sumoylation, thereby facilitating the maintenance of EBV latency.^331^ According to recent research, *TRIM28* has been found to interact with *IFI16*, leading to the suppression of EBV lytic gene expression.^332^ Significantly, the phosphorylation of *TRIM28* impedes its suppressive role on virus latency, consequently promoting virus production.^333^ Moreover, *TRIM28* acts as a repressor of human endogenous retroviruses.^334^ Activation of endogenous retroviruses represents a crucial mechanism in the context of antitumor immune responses triggered by radiotherapy.

## 4 CONCLUSION AND PROSPECTS

In conclusion, *TRIM28*, as a key member of the *TRIM* protein family, plays a pivotal role in maintaining health and contributing to the onset and progression of various diseases. The *TRIM* family of proteins, particularly *TRIM28*, has emerged as a pivotal regulator in the pathogenesis of cancer, influencing a multitude of cellular processes that are critical to tumor development and progression. *TRIM28* is a key member of the *TIF1* family and is characterized by its diverse functional domains, including a RING domain, B‐box motifs, and a coiled‐coil region, which collectively contribute to its versatility in cellular function. These domains enable *TRIM28* to act as a transcriptional coregulator, particularly through its interactions with *KRAB*‐*ZNF* proteins, thereby playing a significant role in chromatin remodeling and gene expression modulation. Additionally, *TRIM28* is deeply involved in the DNA damage response, where its phosphorylation is crucial for the activation and coordination of DNA repair mechanisms. This multifaceted role of *TRIM28* underscores its importance not only in maintaining cellular homeostasis but also in the aberrant processes that lead to oncogenesis. Its involvement in various pathways, including those regulating cell cycle progression, apoptosis, and response to genotoxic stress, makes *TRIM28* a critical player in cancer biology and a potential target for therapeutic intervention. Understanding the complex regulatory functions of *TRIM28* in cancer could provide valuable insights into novel strategies for cancer treatment.

*TRIM28*’s involvement in transcriptional coregulation, particularly with *KRAB*‐*ZNF* proteins, underscores its central role in the intricate processes of chromatin remodeling and gene expression modulation. This partnership is crucial for maintaining chromatin structure, ensuring that gene activity is tightly regulated and responsive to the cellular environment. The *KRAB*‐*ZNF* proteins, a large family of transcriptional repressors, rely on *TRIM28* to recruit additional factors necessary for the establishment of repressive chromatin marks. This interaction not only contributes to the silencing of specific genomic regions but also plays a significant role in preserving genome stability. *TRIM28* to act as a mediator between chromatin dynamics and DNA repair. The dual role of *TRIM28* in gene regulation and DNA repair highlights its critical function in cellular homeostasis and its potential as a therapeutic target in cancer.

In the context of cancer, *TRIM28* displays a highly nuanced and context‐dependent role, characterized by its ability to function either as a protumorigenic factor or as a tumor‐suppressive agent. This duality is influenced by the specific type of cancer and the cellular environment in which it operates. As a protumorigenic factor, *TRIM28* contributes to tumor progression by supporting the maintenance of stem cell properties and promoting cellular characteristics conducive to cancer growth. It influences key aspects of tumor biology, including the regulation of the cancer cell cycle, promotion of cell proliferation, and resistance to apoptotic signals, highlighting its significant impact on cancer development.

Conversely, *TRIM28* can also exhibit tumor‐suppressive properties under certain conditions. Its involvement in the EMT—a process that facilitates cancer metastasis—demonstrates its role in enabling cancer cells to acquire invasive capabilities. Additionally, *TRIM28*’s functions in maintaining stem cell attributes and silencing TEs further illustrate its multifaceted contribution to cancer progression. These roles collectively emphasize the complexity of *TRIM28*’s impact on tumor biology and its potential as a target for therapeutic interventions.

*TRIM28* influences processes such as neurodevelopment, immune regulation, and metabolic control. Dysregulation of *TRIM28* has been linked to a wide range of disorders, including neurodegenerative diseases, autoimmune conditions, and metabolic disorders such as obesity and type 2 diabetes. Its involvement in chronic inflammatory diseases conditions further highlights the importance of *TRIM28* in maintaining genomic stability and cellular function. Additionally, *TRIM28* plays a significant role in viral infections by modulating the host's immune response and influencing viral replication. This antiviral function underscores *TRIM28*’s broader role in host defense mechanisms, making it a critical player not only in disease pathogenesis but also in the body's response to viral challenges.

Given its prominent role in diverse aspects of cancer and other disease development, *TRIM28* represents a promising candidate for targeted therapeutic strategies. Future research should focus on elucidating the precise mechanisms through which *TRIM28* influences disease progression and developing approaches to modulate its activity. By deepening our understanding of *TRIM28*’s functions and interactions, we can potentially uncover innovative treatment options that harness its capabilities to combat cancer more effectively and enhance patient outcomes.

## AUTHOR CONTRIBUTIONS

*Formal analysis, review, design of survey and study, writing, and editing*: Mazaher Maghsoudloo. *Review, formal analysis, writing, and editing*: Khatere Mokhtari. *Formal analysis and review*: Behdokht jamali and Amir Gholamzad. *Survey and design, theory development, supervision, project administration, writing of original draft, reviewing and editing of final manuscript*: Maliheh Entezari and Mehrdad Hashemi. *Concept of the idea, survey and design, theory development, supervision, project administration, writing of original draft, reviewing and editing of final manuscript*: Junjiang Fu. All authors read and approved the contents of the manuscript.

## CONFLICT OF INTEREST STATEMENT

The authors declare no conflict of interest.

## ETHICS STATEMENT

Not applicable.

## Supporting information



Supporting Information

## Data Availability

Not applicable.
