# Supplementary material for: Multifaceted role of TRIM28 in health and disease
Source: MedComm (2020). 2024 Nov 11;5(11):e790. doi: 10.1002/mco2.790 (PMC11554878; doi:10.1002/mco2.790)
Supplement: Supplementary file 1 — Supporting Information [file MCO2-5-e790-s001.docx]

**Multifaceted role of *TRIM28* in health and disease**

**Mazaher Maghsoudloo^1#^, Khatere Mokhtari^2#^, Behdokht jamali^3^, Amir Gholamzad^4^, Maliheh Entezari^4,5*^, Mehrdad Hashemi^5,4*^, Junjiang Fu^1*^**

1. **Key Laboratory of Epigenetics and Oncology, the Research Center for Preclinical Medicine, Southwest Medical University, Luzhou 646000, Sichuan, P.R. China.**
2. **Department of Cellular and Molecular Biology and Microbiology, Faculty of Biological Science and Technology, University of Isfahan, Isfahan, Iran.**
3. **Department of microbiology and genetic, kherad Institute of higher education, Busheher, lran.**
4. **Farhikhtegan Medical Convergence Sciences Research Center, Farhikhtegan Hospital Tehran Medical Sciences, Islamic Azad University, Tehran, Iran.**
5. **Department of Genetics, Faculty of Advanced Science and Technology, Tehran Medical Sciences, Islamic Azad University, Tehran, Iran.**

**^#^ These authors contributed equally to this work and share the first authorship.**

***Corresponding Authors:**

**Junjiang Fu (**[**fujunjiang@swmu.edu.cn**](mailto:fujunjiang@swmu.edu.cn)**)**

**Key Laboratory of Epigenetics and Oncology, the Research Center for Preclinical Medicine, Southwest Medical University, Luzhou 646000, Sichuan, P.R. China.**

**Merhrdad Hashemi (****[drmehashemi@iautmu.ac.ir](mailto:drmehashemi@iautmu.ac.ir))**

**Department of Genetics, Faculty of Advanced Science and Technology, Tehran Medical Sciences, Islamic Azad University, Tehran, Iran.**

**Maliheh Entezari (**[**mentezari@iautmu.ac.ir**](mailto:mentezari@iautmu.ac.ir)**)**

**Farhikhtegan Medical Convergence Sciences Research Center, Farhikhtegan Hospital Tehran Medical Sciences, Islamic Azad University, Tehran, Iran.**

**Table S1.** *TRIM* family proteins associated with unfavorable prognostic outcomes.

| TRIM family | AML | HCC | CRC | Squamous arcinoma | Lung carcinoma | Glioblastoma | Pancreatic arcinoma | OC | Breast carcinoma | NPC | RCC | melanoma | GBC | Gastric carcinoma | HEC | TGCT | UCB | PTC | glioma | Cervical cancer | prostate cancer | Osteosarcoma | Ref.s |
| --- | --- | --- | --- | --- | --- | --- | --- | --- | --- | --- | --- | --- | --- | --- | --- | --- | --- | --- | --- | --- | --- | --- | --- |
| *TRIM1* |  |  | ***** |  |  |  |  |  |  |  |  |  |  |  |  |  |  |  |  |  |  |  | ^1^ |
| *TRIM2* |  |  | ***** |  |  |  |  |  |  |  |  |  |  |  |  |  |  |  |  |  |  |  | ^1^ |
| *TRIM3* |  | * |  |  |  |  |  |  | * |  |  |  |  | * |  |  |  |  |  |  |  |  | ^2-4^ |
| *TRIM5* |  | ***** |  |  |  |  |  |  |  |  |  |  |  |  |  |  |  |  |  |  |  |  | ^2^ |
| *TRIM6* |  |  | ***** |  |  |  |  |  |  |  |  |  |  |  |  |  |  |  |  |  |  |  | ^1^ |
| *TRIM7* |  |  |  |  |  |  |  |  |  |  |  |  |  |  |  |  |  |  |  |  |  | ***** | ^5^ |
| *TRIM8* |  |  |  |  |  | ***** |  |  |  |  |  |  |  |  |  |  |  |  |  |  |  |  | ^6^ |
| *TRIM11* |  | ***** |  |  | ***** |  | ***** |  |  | ***** |  |  |  |  |  |  |  |  |  | * |  |  | ^7-12^ |
| *TRIM13* |  |  | ***** |  |  |  |  |  |  |  |  |  |  |  |  |  |  |  |  |  |  |  | ^1^ |
| *TRIM14* |  |  |  |  |  | ***** |  |  |  |  |  |  |  | ***** |  |  |  | ***** |  |  |  |  | ^13-15^ |
| *TRIM15* |  |  |  |  | ***** |  | ***** |  |  |  |  |  | ***** |  |  |  |  |  |  |  |  |  | ^16-18^ |
| *TRIM16* |  | ***** |  |  |  | ***** |  |  | ***** |  |  |  |  |  |  |  |  |  |  |  |  |  | ^19, 20^ |
| *TRIM17* |  |  |  |  |  | ***** |  |  |  |  |  |  |  |  |  |  |  |  |  |  |  |  | ^21^ |
| *TRIM21* |  | ***** |  |  |  | ***** |  |  |  |  |  |  |  | ***** |  |  |  |  |  |  |  |  | ^2, 22, 23^ |
| *TRIM22* |  |  |  |  |  |  |  |  |  |  |  |  |  |  |  |  |  |  |  |  |  | ***** | ^24^ |
| *TRIM23* |  |  | ***** |  | ***** |  |  |  |  |  |  |  |  | ***** |  |  |  |  |  |  |  |  | ^25-27^ |
| *TRIM24* |  | ***** | ***** |  |  |  |  |  |  |  |  |  |  |  |  |  |  |  |  |  |  |  | ^28, 29^ |
| *TRIM26* |  | ***** | ***** |  |  |  |  |  |  |  |  |  |  |  |  |  |  |  |  |  |  |  | ^1, 30^ |
| *TRIM27* |  | ***** | ***** |  |  |  |  |  |  |  | ***** | ***** |  |  |  |  |  |  |  |  |  |  | ^31-34^ |
| *TRIM28* |  | ***** | ***** | ***** | ***** |  |  |  |  |  |  |  |  | * |  |  |  |  | * | * | * |  | ^35-43^ |
| *TRIM29* |  |  |  |  |  |  |  |  | ***** |  |  |  |  | ***** |  |  |  |  |  | * |  |  | ^44-46^ |
| *TRIM31* |  | ***** |  |  |  |  |  |  | ***** |  |  |  | ***** |  |  |  |  |  |  |  |  |  | ^47-49^ |
| *TRIM32* |  | ***** |  |  |  |  |  |  |  |  |  |  |  | ***** |  |  |  |  |  |  |  |  | ^2, 50, 51^ |
| *TRIM35* |  |  | ***** |  | ***** |  |  |  | ***** |  |  |  |  |  |  |  |  |  |  |  |  |  | ^1, 52^ |
| *TRIM36* |  |  |  |  |  |  | ***** |  |  |  |  |  |  |  |  |  |  |  |  |  |  |  | ^53^ |
| *TRIM37* |  | ***** |  |  |  |  | ***** |  |  |  | ***** |  |  |  |  |  |  |  |  |  |  |  | ^7, 35, 54, 55^ |
| *TRIM39* |  |  | ***** |  |  |  |  |  | ***** |  |  |  |  |  |  |  |  |  |  |  |  |  | ^56^ |
| *TRIM44* |  | ***** |  |  |  |  |  |  | ***** |  | ***** |  |  | ***** | ***** | ***** |  |  |  |  |  |  | ^2, 53, 54, 57-60^ |
| *TRIM45* |  | ***** |  |  |  |  |  |  |  |  |  |  |  |  |  |  |  |  |  |  |  |  | ^61^ |
| *TRIM46* |  |  |  |  | ***** |  |  |  |  |  |  |  |  |  |  |  |  |  |  |  |  |  | ^62^ |
| *TRIM47* |  | ***** | ***** |  |  |  | ***** |  | ***** |  |  |  |  | ***** |  |  |  |  |  |  |  |  | ^1, 2, 63-65^ |
| *TRIM50* |  | ***** |  |  |  |  | ***** | ***** |  |  |  |  |  |  |  |  |  |  |  |  |  |  | ^61, 66^ |
| *TRIM54* |  |  |  |  |  |  |  |  |  |  |  |  |  | ***** |  |  |  |  |  |  |  |  | ^67^ |
| *TRIM55* |  |  | ***** |  |  |  |  |  |  |  |  |  |  |  |  |  |  |  |  |  |  |  | ^1^ |
| *TRIM56* |  |  |  |  | ***** |  |  |  |  |  |  |  |  |  |  |  |  |  |  |  |  |  | ^68^ |
| *TRIM58* |  |  | ***** |  |  |  |  |  |  |  |  |  |  |  |  |  |  |  |  |  |  |  | ^69^ |
| *TRIM59* |  | ***** |  |  |  |  | ***** | ***** | ***** |  |  |  |  |  |  |  |  |  |  |  |  |  | ^61, 65, 70-72^ |
| *TRIM62* | ***** |  |  |  |  |  |  |  | ***** |  |  |  |  |  |  |  |  |  |  |  |  |  | ^73, 74^ |
| *TRIM65* |  |  |  |  |  |  |  |  |  |  |  |  |  |  |  |  | ***** |  |  |  |  |  | ^75^ |
| *TRIM66* |  |  |  |  | ***** |  |  |  |  |  |  |  |  |  |  |  |  |  |  |  |  |  | ^76^ |
| *TRIM72* |  | ***** |  |  |  |  |  |  |  |  |  |  |  |  |  |  |  |  |  |  |  |  | ^2^ |

*, **OC**: ovarian cancer, **TGCT**: testicular germ cell tumors, **PTC**: papillary thyroid cancer, **UCB**: urothelium carcinoma, **HCC**: hepatocellular carcinoma, **AML**: acute myelocytic leukemia, **CRC**: colorectal cancer, **NPC**: nasopharyngeal carcinoma, **HEC**: human esophageal cancer, **RCC**: renal cell carcinoma

**
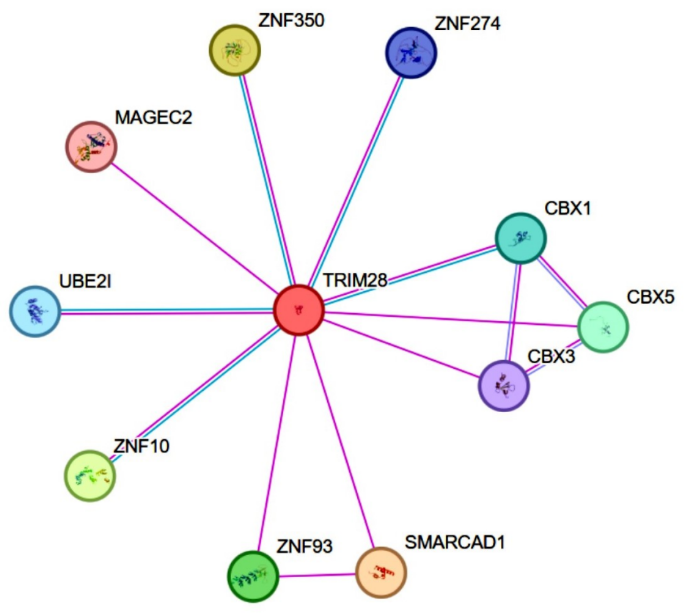
**

**Figure S1.** The PPI network of the *TRIM28* reconstructed using the STRING online database (<https://string-db.org/>).  For the interaction resources parameter in STRING, we considered experiments and databases, setting the minimum required interaction score to 0.7.

**References**

1. Qiu Y, Liu P, Ma X, et al. TRIM50 acts as a novel Src suppressor and inhibits ovarian cancer progression. *Biochim Biophys Acta Mol Cell Res*. 2019;1866(9):1412-1420.

2. Guo P, Ma X, Zhao W, et al. TRIM31 is upregulated in hepatocellular carcinoma and promotes disease progression by inducing ubiquitination of TSC1-TSC2 complex. *Oncogene*. 2018;37(4):478-488.

3. Guo Y, Li Q, Zhao G, et al. Loss of TRIM31 promotes breast cancer progression through regulating K48- and K63-linked ubiquitination of p53. *Cell Death Dis*. 2021;12(10):945.

4. Fujimura T, Inoue S, Urano T, et al. Increased Expression of Tripartite Motif (TRIM) 47 Is a Negative Prognostic Predictor in Human Prostate Cancer. *Clin Genitourin Cancer*. 2016;14(4):298-303.

5. Liu B, Li X, Liu F, et al. Expression and Significance of TRIM 28 in Squamous Carcinoma of Esophagus. *Pathol Oncol Res*. 2019;25(4):1645-1652.

6. Xia Y, Wei Z, Huang W, Wei X, He Y. Trim47 overexpression correlates with poor prognosis in gastric cancer. *Neoplasma*. 2021;68(2):307-316.

7. Wu L, Yin X, Jiang K, et al. Comprehensive profiling of the TRIpartite motif family to identify pivot genes in hepatocellular carcinoma. *Cancer Med*. 2022;11(7):1712-1731.

8. Ma X, Ma X, Qiu Y, et al. TRIM50 suppressed hepatocarcinoma progression through directly targeting SNAIL for ubiquitous degradation. *Cell Death Dis*. 2018;9(6):608.

9. Xiong D, Jin C, Ye X, et al. TRIM44 promotes human esophageal cancer progression via the AKT/mTOR pathway. *Cancer Sci*. 2018;109(10):3080-3092.

10. Kimura N, Yamada Y, Takayama KI, et al. Androgen-responsive tripartite motif 36 enhances tumor-suppressive effect by regulating apoptosis-related pathway in prostate cancer. *Cancer Sci*. 2018;109(12):3840-3852.

11. Chen L, Chen DT, Kurtyka C, et al. Tripartite motif containing 28 (Trim28) can regulate cell proliferation by bridging HDAC1/E2F interactions. *J Biol Chem*. 2012;287(48):40106-18.

12. Liu W, Wu Z, Wang L, Wang Q, Sun X, Niu S. Knockdown of TRIM11 suppresses cell progression and apoptosis of cervical cancer cells via PI3K/AKT pathway. *Am J Transl Res*. 2021;13(9):10328-10340.

13. Wei WS, Chen X, Guo LY, et al. TRIM65 supports bladder urothelial carcinoma cell aggressiveness by promoting ANXA2 ubiquitination and degradation. *Cancer Lett*. 2018;435:10-22.

14. Farhadi J, Goshayeshi L, Motavalizadehkakhky A, Mehrzad J, Mehrad-Majd H. Decreased expression of TRIM3 gene predicts a poor prognosis in gastric cancer. *J Gastrointest Cancer*. 2022;53(1):179-186.

15. Kim PY, Tan O, Liu B, et al. High TDP43 expression is required for TRIM16-induced inhibition of cancer cell growth and correlated with good prognosis of neuroblastoma and breast cancer patients. *Cancer Lett*. 2016;374(2):315-23.

16. Li R, Zhu L, Peng Y, Zhang X, Dai C, Liu D. TRIM50 Suppresses Pancreatic Cancer Progression and Reverses the Epithelial-Mesenchymal Transition via Facilitating the Ubiquitous Degradation of Snail1. *Front Oncol*. 2021;11:695740.

17. Ping M, Wang S, Guo Y, Jia J. TRIM21 improves apatinib treatment in gastric cancer through suppressing EZH1 stability. *Biochem Biophys Res Commun*. 2022;586:177-184.

18. Lu K, Sui Y, Fu L. Identification of TRIM56 as a Potential Biomarker for Lung Adenocarcinoma. *Cancer Manag Res*. 2021;13:2201-2213.

19. Chen Y, Li L, Qian X, Ge Y, Xu G. High expression of TRIM11 correlates with poor prognosis in patients with hepatocellular carcinoma. *Clin Res Hepatol Gastroenterol*. 2017;41(2):190-196.

20. Cao H, Li Y, Chen L, et al. Tripartite motif-containing 54 promotes gastric cancer progression by upregulating K63-linked ubiquitination of filamin C. *Asia Pac J Clin Oncol*. 2022;18(6):669-677.

21. Micale L, Fusco C, Fontana A, et al. TRIM8 downregulation in glioma affects cell proliferation and it is associated with patients survival. *BMC Cancer*. 2015;15:470.

22. Li R, Weng L, Liu B, et al. TRIM59 predicts poor prognosis and promotes pancreatic cancer progression via the PI3K/AKT/mTOR-glycolysis signaling axis. *J Cell Biochem*. 2020;121(2):1986-1997.

23. Xiao S, Yu J, Yuan X, Chen Q. Identification of a tripartite motif family gene signature for predicting the prognosis of patients with glioma. *Am J Transl Res*. 2022;14(3):1535-1550.

24. Xia Y, Zhao J, Yang C. Identification of key genes and pathways for melanoma in the TRIM family. *Cancer Med*. 2020;9(23):8989-9005.

25. Zheng D, Zhang Y, Xia Y, Cheng F. A Novel Gene Signature of Tripartite Motif Family for Predicting the Prognosis in Kidney Renal Clear Cell Carcinoma and Its Association With Immune Cell Infiltration. *Front Oncol*. 2022;12:840410.

26. Han X, Huang C, Qu X, et al. Tripartite motif-containing 15 overexpression in non-small cell lung cancer is associated with poor patient prognoses. *J Cancer*. 2019;10(4):843-852.

27. Wang F, Ruan L, Yang J, Zhao Q, Wei W. TRIM14 promotes the migration and invasion of gastric cancer by regulating epithelial‑to‑mesenchymal transition via activation of AKT signaling regulated by miR‑195‑5p. *Oncol Rep*. 2018;40(6):3273-3284.

28. Liu M, Zhang X, Cai J, et al. Downregulation of TRIM58 expression is associated with a poor patient outcome and enhances colorectal cancer cell invasion. *Oncol Rep*. 2018;40(3):1251-1260.

29. Yang J, Ye J, Ma T, et al. Tripartite motif-containing protein 11 promotes hepatocellular carcinogenesis through ubiquitin-proteasome-mediated degradation of pleckstrin homology domain leucine-rich repeats protein phosphatase 1. *Hepatology*. 2022;76(3):612-629.

30. Zhan W, Han T, Zhang C, et al. TRIM59 Promotes the Proliferation and Migration of Non-Small Cell Lung Cancer Cells by Upregulating Cell Cycle Related Proteins. *PLoS One*. 2015;10(11):e0142596.

31. Liu X, Huang Y, Yang D, et al. Overexpression of TRIM24 is associated with the onset and progress of human hepatocellular carcinoma. *PLoS One*. 2014;9(1):e85462.

32. Tan G, Xie B, Yu N, et al. TRIM37 overexpression is associated with chemoresistance in hepatocellular carcinoma via activating the AKT signaling pathway. *Int J Clin Oncol*. 2021;26(3):532-542.

33. Tantai J, Pan X, Chen Y, Shen Y, Ji C. TRIM46 activates AKT/HK2 signaling by modifying PHLPP2 ubiquitylation to promote glycolysis and chemoresistance of lung cancer cells. *Cell Death Dis*. 2022;13(3):285.

34. Han Y, Tan Y, Zhao Y, et al. TRIM23 overexpression is a poor prognostic factor and contributes to carcinogenesis in colorectal cancer. *J Cell Mol Med*. 2020;24(10):5491-5500.

35. Quintas-Cardama A, Zhang N, Qiu YH, et al. Loss of TRIM62 expression is an independent adverse prognostic factor in acute myeloid leukemia. *Clin Lymphoma Myeloma Leuk*. 2015;15(2):115-127 e15.

36. Ma Y, Dai HY, Zhang F, Zhao D. TRIM66 expression in non-small cell lung cancer: A new predictor of prognosis. *Cancer Biomark*. 2017;20(3):309-315.

37. Liu Y, Dong Y, Zhao L, Su L, Diao K, Mi X. TRIM59 overexpression correlates with poor prognosis and contributes to breast cancer progression through AKT signaling pathway. *Mol Carcinog*. 2018;57(12):1792-1802.

38. Li F, Wang Z, Lu G. TRIM28 promotes cervical cancer growth through the mTOR signaling pathway. *Oncol Rep*. 2018;39(4):1860-1866.

39. Fong KW, Zhao JC, Song B, Zheng B, Yu J. TRIM28 protects TRIM24 from SPOP-mediated degradation and promotes prostate cancer progression. *Nat Commun*. 2018;9(1):5007.

40. Nyberg WA, Velasquez-Pulgarin DA, He T, et al. The bromodomain protein TRIM28 controls the balance between growth and invasiveness in melanoma. *EMBO Rep*. 2023;24(1):e54944.

41. Su C, Li H, Gao W. TRIM28 is overexpressed in glioma and associated with tumor progression. *Onco Targets Ther*. 2018;11:6447-6458.

42. Yokoe T, Toiyama Y, Okugawa Y, et al. KAP1 is associated with peritoneal carcinomatosis in gastric cancer. *Ann Surg Oncol*. 2010;17(3):821-8.

43. Fitzgerald S, Sheehan KM, O'Grady A, et al. Relationship between epithelial and stromal TRIM28 expression predicts survival in colorectal cancer patients. *J Gastroenterol Hepatol*. 2013;28(6):967-74.

44. Chen W, Lu C, Hong J. TRIM15 Exerts Anti-Tumor Effects Through Suppressing Cancer Cell Invasion in Gastric Adenocarcinoma. *Med Sci Monit*. 2018;24:8033-8041.

45. Zhao Z, Wang Y, Yun D, et al. TRIM21 overexpression promotes tumor progression by regulating cell proliferation, cell migration and cell senescence in human glioma. *Am J Cancer Res*. 2020;10(1):114-130.

46. Xu R, Hu J, Zhang T, Jiang C, Wang HY. TRIM29 overexpression is associated with poor prognosis and promotes tumor progression by activating Wnt/beta-catenin pathway in cervical cancer. *Oncotarget*. 2016;7(19):28579-91.

47. Liu Y, Tao S, Liao L, et al. TRIM25 promotes the cell survival and growth of hepatocellular carcinoma through targeting Keap1-Nrf2 pathway. *Nat Commun*. 2020;11(1):348.

48. Yamada Y, Kimura N, Takayama KI, et al. TRIM44 promotes cell proliferation and migration by inhibiting FRK in renal cell carcinoma. *Cancer Sci*. 2020;111(3):881-890.

49. Qi ZX, Cai JJ, Chen LC, et al. TRIM28 as an independent prognostic marker plays critical roles in glioma progression. *J Neurooncol*. 2016;126(1):19-26.

50. Yao Y, Liu Z, Guo H, et al. Elevated TRIM23 expression predicts poor prognosis in Chinese gastric cancer. *Pathol Res Pract*. 2018;214(12):2062-2068.

51. Kosaka Y, Inoue H, Ohmachi T, et al. Tripartite motif-containing 29 (TRIM29) is a novel marker for lymph node metastasis in gastric cancer. *Ann Surg Oncol*. 2007;14(9):2543-9.

52. Liang M, Wang L, Sun Z, et al. E3 ligase TRIM15 facilitates non-small cell lung cancer progression through mediating Keap1-Nrf2 signaling pathway. *Cell Commun Signal*. 2022;20(1):62.

53. Ma J, Wang L, Li J, et al. Swainsonine Inhibits Invasion and the EMT Process in Esophageal Carcinoma Cells by Targeting Twist1. *Oncol Res*. 2018;26(8):1207-1213.

54. Xiao C, Zhang W, Hua M, et al. TRIM27 interacts with Ikappabalpha to promote the growth of human renal cancer cells through regulating the NF-kappaB pathway. *BMC Cancer*. 2021;21(1):841.

55. Pan Y, Zhang R, Chen H, Chen W, Wu K, Lv J. Expression of Tripartite Motif-Containing Proteactiin 11 (TRIM11) is Associated with the Progression of Human Prostate Cancer and is Downregulated by MicroRNA-5193. *Med Sci Monit*. 2019;25:98-106.

56. Wang FQ, Han Y, Yao W, Yu J. Prognostic relevance of tripartite motif containing 24 expression in colorectal cancer. *Pathol Res Pract*. 2017;213(10):1271-1275.

57. Ye R, AiErken N, Kuang X, et al. Tripartite motif-containing 3 (TRIM3) enhances ER signaling and confers tamoxifen resistance in breast cancer. *Oncogenesis*. 2021;10(9):60.

58. Ogura T, Azuma K, Takeiwa T, et al. TRIM39 is a poor prognostic factor for patients with estrogen receptor-positive breast cancer and promotes cell cycle progression. *Pathol Int*. 2022;72(2):96-106.

59. Sun W, Wang Y, Li D, Wu Y, Ji Q, Sun T. Tripartite motif containing 14: An oncogene in papillary thyroid carcinoma. *Biochem Biophys Res Commun*. 2020;521(2):360-367.

60. Wang C, Xu J, Fu H, et al. TRIM32 promotes cell proliferation and invasion by activating beta-catenin signalling in gastric cancer. *J Cell Mol Med*. 2018;22(10):5020-5028.

61. Wang Y, He D, Yang L, et al. TRIM26 functions as a novel tumor suppressor of hepatocellular carcinoma and its downregulation contributes to worse prognosis. *Biochem Biophys Res Commun*. 2015;463(3):458-65.

62. Zhang Y, Du H, Li Y, Yuan Y, Chen B, Sun S. Elevated TRIM23 expression predicts cisplatin resistance in lung adenocarcinoma. *Cancer Sci*. 2020;111(2):637-646.

63. Wang J, Fang Y, Liu T. TRIM32 Promotes the Growth of Gastric Cancer Cells through Enhancing AKT Activity and Glucose Transportation. *Biomed Res Int*. 2020;2020:4027627.

64. Sun Y, Ren D, Yang C, et al. TRIM15 promotes the invasion and metastasis of pancreatic cancer cells by mediating APOA1 ubiquitination and degradation. *Biochim Biophys Acta Mol Basis Dis*. 2021;1867(11):166213.

65. Kawabata H, Azuma K, Ikeda K, et al. TRIM44 Is a Poor Prognostic Factor for Breast Cancer Patients as a Modulator of NF-kappaB Signaling. *Int J Mol Sci*. 2017;18(9)

66. Hu J, Ding X, Tian S, et al. TRIM39 deficiency inhibits tumor progression and autophagic flux in colorectal cancer via suppressing the activity of Rab7. *Cell Death Dis*. 2021;12(4):391.

67. Kashimoto K, Komatsu S, Ichikawa D, et al. Overexpression of TRIM44 contributes to malignant outcome in gastric carcinoma. *Cancer Sci*. 2012;103(11):2021-6.

68. Lott ST, Chen N, Chandler DS, et al. DEAR1 is a dominant regulator of acinar morphogenesis and an independent predictor of local recurrence-free survival in early-onset breast cancer. *PLoS Med*. 2009;6(5):e1000068.

69. Wang Y, Zhou Z, Wang X, et al. TRIM59 Is a Novel Marker of Poor Prognosis and Promotes Malignant Progression of Ovarian Cancer by Inducing Annexin A2 Expression. *Int J Biol Sci*. 2018;14(14):2073-2082.

70. Zhang R, Li SW, Liu L, Yang J, Huang G, Sang Y. TRIM11 facilitates chemoresistance in nasopharyngeal carcinoma by activating the beta-catenin/ABCC9 axis via p62-selective autophagic degradation of Daple. *Oncogenesis*. 2020;9(5):45.

71. Azuma K, Ikeda K, Suzuki T, Aogi K, Horie-Inoue K, Inoue S. TRIM47 activates NF-kappaB signaling via PKC-epsilon/PKD3 stabilization and contributes to endocrine therapy resistance in breast cancer. *Proc Natl Acad Sci U S A*. 2021;118(35)

72. Chen S, He Z, Zhu C, et al. TRIM37 Mediates Chemoresistance and Maintenance of Stemness in Pancreatic Cancer Cells via Ubiquitination of PTEN and Activation of the AKT-GSK-3beta-beta-Catenin Signaling Pathway. *Front Oncol*. 2020;10:554787.

73. Yanagi T, Watanabe M, Hata H, et al. Loss of TRIM29 Alters Keratin Distribution to Promote Cell Invasion in Squamous Cell Carcinoma. *Cancer Res*. 2018;78(24):6795-6806.

74. Tan P, Ye Y, He L, et al. TRIM59 promotes breast cancer motility by suppressing p62-selective autophagic degradation of PDCD10. *PLoS Biol*. 2018;16(11):e3000051.

75. Miao C, Liang C, Li P, et al. TRIM37 orchestrates renal cell carcinoma progression via histone H2A ubiquitination-dependent manner. *J Exp Clin Cancer Res*. 2021;40(1):195.

76. Zhang J, Xu Z, Yu B, Xu J, Yu B. Tripartite motif containing 35 contributes to the proliferation, migration, and invasion of lung cancer cells in vitro and in vivo. *Biosci Rep*. 2020;40(4)
